# Biotechnology-driven extraction and characterisation of Chitosan from the West African river prawn (*Macrobrachium vollenhovenii*) and American Cockroach (*Periplaneta americana*) using a modified approach

**DOI:** 10.1371/journal.pone.0349133

**Published:** 2026-05-28

**Authors:** Adebowale E. Aderogba, Olukemi K. Amodu, Oluwayemi Joshua Bamikole, Babajide A. Adedeji, Miles-Dei B. Olufeagba

**Affiliations:** 1 Public Health Biotechnology Program, Genetics and Molecular Sciences Unit, Institute of Child Health, College of Medicine, University of Ibadan, Ibadan, Nigeria; 2 Department of Microbiology, Modibbo Adama University of Technology, Yola, Nigeria; Lusofona University of Humanities and Technologies: Universidade Lusofona de Humanidades e Tecnologias, PORTUGAL

## Abstract

Chitosan is a natural biopolymer derived from the deacetylation of chitin, a structural polysaccharide abundantly found in the shells and exoskeletons of crustaceans, insects, and other arthropods. Its unique physicochemical properties have led to widespread applications in biomedical, pharmaceutical, agricultural, and industrial sectors. In Nigeria, abundant freshwater crustaceans and insects remain underutilized as sources of chitosan. This study evaluated the potential of West African river prawns (*Macrobrachium vollenhovenii*) and American cockroaches (*Periplaneta americana*) as local chitosan sources using a modified chemical extraction process. Exoskeletal materials were pretreated, demineralized, deproteinized, and subjected to autoclave assisted alkaline deacetylation (50% NaOH, 121 °C, 15 psi, 30 min). Chitin yields were 29.53% for prawns and 17.78% for cockroaches, while chitosan yields were 28.13% and 11.56%, respectively. Fourier Transform Infrared Spectroscopy confirmed characteristic functional groups of chitosan in both sources. The degree of deacetylation (DD) was 68.79% for prawn-derived chitosan and 81.21% for cockroach-derived chitosan, indicating effective conversion of N-acetyl. D-glucosamine to D-glucosamine units. These findings demonstrate that both species are viable alternative sources for chitosan production, with pressurized deacetylation enhancing yield and polymer quality. This approach provides a scalable, reproducible strategy for sustainable chitosan extraction in Nigeria, supporting potential applications in biotechnology, medicine, and industry.

## Introduction

Chitosan is a natural biopolymer, composed of β-(1–4)-linked D-glucosamine and N-acetyl-D glucosamine units [1]. It is derived from the deacetylation of chitin, a structural polysaccharide abundantly present in the exoskeletons of crustaceans, insects, fungi and other organisms [2,3]. Owing to its biodegradability, biocompatibility, and non-toxicity, chitosan has garnered significant attention globally. The demand for chitosan is increasing with its market value projected to reach $15.1 billion by 2030 [4], reflecting expanding applications, including biomedical and pharmaceutical sciences, food technology, agriculture, water purification, textile engineering, and environmental protection [5–7].

Industrial production of chitosan traditionally relies on utilising crustacean shell waste, employing sequential chemical treatments involving: demineralization, deproteinization, and deacetylation [8, 9]. Among these steps, deacetylation represents the most time-intensive and rate-limiting step, with conventional alkaline processes typically requiring prolonged reaction times ranging from 6 to over 24 hours to achieve high degrees of deacetylation (DD) and acceptable purity [10]. These extended processing durations increase energy consumption, increase production costs, and limit scalability. Therefore, the development of alternative deacetylation strategies capable of reducing reaction time while maintaining or improving chitosan yield and quality remains a critical objective in chitosan processing research.

Beyond process-related limitation, reliance on marine crustaceans is constrained by the seasonality of harvesting cycles and geographical variability [11], presenting freshwater species and insects as promising alternative sources owing to their local availability, rapid biomass regeneration, and favourable chitin composition [12,13]. Recent studies have increasingly explored insect-derived chitin.

In Nigeria, both insects and crustaceans are widely distributed and readily accessible [14], yet remain underutilized for chitosan production. The African giant river prawn (*Macrobrachium vollenhovenii*), widely distributed across West Africa and harvested year-round, has not been extensively evaluated for its chitosan extraction potential. Similarly, the American cockroach (*Periplaneta americana*), though commonly regarded as a pest, possesses a chitin rich exoskeleton and represents an unconventional but potentially viable source of chitosan. Despite their availability, studies investigating these species using modified or accelerated deacetylation techniques are limited, particularly within the West African context.In this context, the present study is motivated by: (i) the need to reduce prolonged deacetylation times associated with conventional alkaline methods, and (ii) the limited exploration of locally abundant arthropods in Nigeria as viable chitosan sources. Chitosan was therefore extracted from locally sourced *M. vollenhovenii* and *P. americana* using a modified pressurized alkaline deacetylation approach. The study comparatively evaluates extraction yield, functional group characterization, and degree of deacetylation, demonstrating that high-yield chitosan can be obtained from locally sourced Nigerian arthropods through a scalable and time-efficient method.

## Methodology

### Sample collection and preparation

This study was conducted as an exploratory, laboratory-based experimental investigation to assess the feasibility of a modified pressurised alkaline deacetylation approach for chitosan extraction.

### Sample selection

A total of fifty-two (52) adult cockroaches (*Periplaneta americana*) were collected from residential areas in Ibadan, Nigeria, and maintained in an insectary under controlled conditions (27  ± 2 °C, continuous darkness) for 30–90 days with a diet of dry bread, fruits, and water. In addition, twenty (20) African river prawns (*Macrobrachium vollenhovenii*), comprising both adults and juveniles, were obtained from Eleyele Lake, Ibadan. Sample size was determined based on the minimum exoskeletal biomass required for efficient chitin extraction, chitosan yield determination, and subsequent physicochemical characterisation. Preliminary processing trials indicated that the selected numbers provided sufficient and reproducible material for the full analytical workflow. Given the exploratory and process-optimization nature of the study, no formal statistical power calculation was performed, as the objective was not hypothesis testing but to evaluate extraction feasibility and material properties.

Cockroaches were euthanized in accordance with standard invertebrate ethical guidelines. Specimens were first immobilized by cold anesthesia on ice until unresponsive, and subsequently frozen at −20°C for a minimum of 30 minutes to ensure humane and complete euthanasia. Prawns were sacrificed by immersion in crushed ice slurry for 20 minutes until cessation of movement, a standard and ethically accepted approach for crustaceans

Exoskeletal materials (cockroach cuticles and prawn shells) were manually separated, washed thoroughly with distilled water to remove adhering impurities, and oven-dried at 35–37 °C until constant weight. The dried materials were subsequently ground into fine powder for use in chitin extraction.

### Ethical Approval

Approval was granted (AD 13/479/035) by the Oyo State Ethical Board.

### Chitosan Extraction technique

Chitosan extraction technique was performed following the method described by Sagheer [15], with modifications (Table 1).

**Table 1:** Demineralization and Deproteinization Treatment Conditions for Cockroach and Prawn Exoskeletons.

Exoskeleton Sample	Sample Initial Dry Weight (DW)g	Pre-treatment	Treatment Condition for Chitin
American Cockroach	9	Degutting, washing, grinding, drying; 1M HCL (Rm. Temp, 12hrs)	1M HCL (Rm. Temp, 3hrs), 1M NaOH (100℃, 3hrs)
African River Prawn	15	Deshelling, washing, grinding, drying	1M HCL (Rm. Temp, 24hrs), 1M NaOH (100℃, 3hrs)

The exoskeleton (cockroach cuticles and prawn shells) was treated with 1 M hydrochloric acid (HCl; Sigma-Aldrich) at a liquid-to-solid ratio of 20:1. The acid treatment facilitated the removal of mineral components. The mixture (exoskeleton and acid) was passed through a 20 µm cloth sieve. The obtained exoskeletal residue was washed repeatedly with distilled water until the pH of the residue became neutral. The exoskeleton was dried to a constant weight at oven temperature (35-37°C). This procedure is the demineralization step. The variation in pre-treatment and demineralization durations between cockroach and prawn samples was based on preliminary optimization and structural differences in exoskeleton composition. Prawn shells possess higher mineral content and thicker calcified matrices compared to cockroach cuticles, necessitating longer acid exposure for effective demineralization. These species-specific adjustments were implemented to ensure complete removal of inorganic components prior to deproteinization and deacetylation.

The dried exoskeleton was deproteinized by treating the demineralized exoskeleton with 1 M NaOH; Sigma-Aldrich) at a liquid-to-solid ratio of 20:1. The mixture was passed through a 20 µm cloth sieve. The residue was washed with distilled water until the pH became neutral, and the resulting chitin was dried to a constant weight at oven temperature (35-37 °C). The chitin yield was calculated as:


$$\begin{array}{c} (\text{Weight of Chitin }/\text{ Weight of Starting Material})\;\times \text{ 100}. \end{array}$$

The obtained chitin was treated with 50% (w/v) sodium hydroxide solution (NaOH; Sigma-Aldrich) under pressurized alkaline conditions as indicated in Table 2, using a standard laboratory autoclave. This treatment facilitated the removal of acetyl groups, resulting in the formation of chitosan. Following treatment, the chitosan were washed with distilled water until neutral pH was achieved and then dried to a constant weight at oven temperature (35–37°C). The chitosan yield was calculated as:

**Table 2:** Deacetylation Treatment Conditions for Obtained Cockroach and Prawn Chitin.

Chitin Sample	Sample Initial Dry Weight (DW)g	Treatment Condition for Chitosan
American Cockroach	9	50% NaOH (121℃, 15 psi, 30mins)
African River Prawn	15	50% NaOH (121℃, 15 psi, 30mins)


$$\begin{array}{c} (\text{Weight of Chitosan }/\text{ Weight of Starting Material})\;\times \text{ 100} \end{array}$$

All yield calculations and degree of deacetylation values were determined from single experimental runs and are presented descriptively.

Fourier Transform Infrared Spectroscopy (FTIR) analysis was performed using a PerkinElmer FT-IR Spectrophotometer (UATR Two, PerkinElmer Inc., Waltham, MA, USA) over the wavelength range of 4000–400 cm^−^¹. FTIR spectra analysis were analyzed by identifying characteristic absorption bands and comparing peak positions with reference chitosan spectra reported in the literature (ref). The reduction in the amide bands was evaluated to confirm successful deacetylation of chitin.. Degree of deacetylation (DD) of the obtained chitosan was calculated the absorbance band ratios, using the equation described by Sabnis and Block [16].


$$\begin{array}{c} 𝐃𝐃\;(\%)\;=\text{ 97}.\text{67 }-\;[\text{26}.\text{486 x }(\text{A1658}/\text{A3450})]. \end{array}$$

## Results

**Fig 1:**
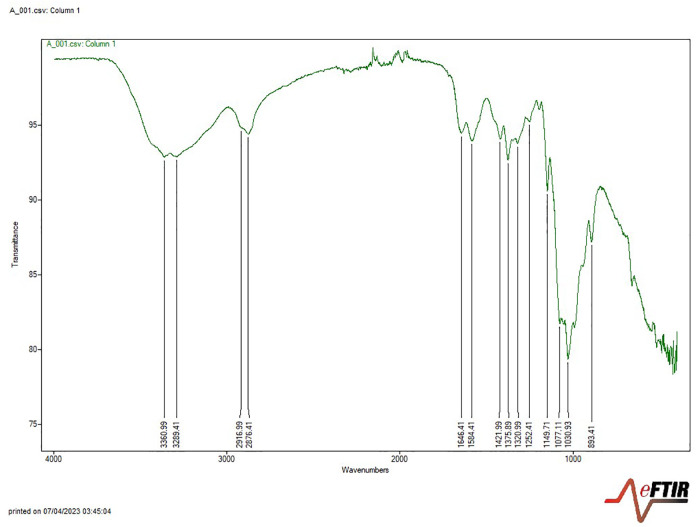
FTIR spectra of extracted cockroach chitosan.

**Fig 2:**
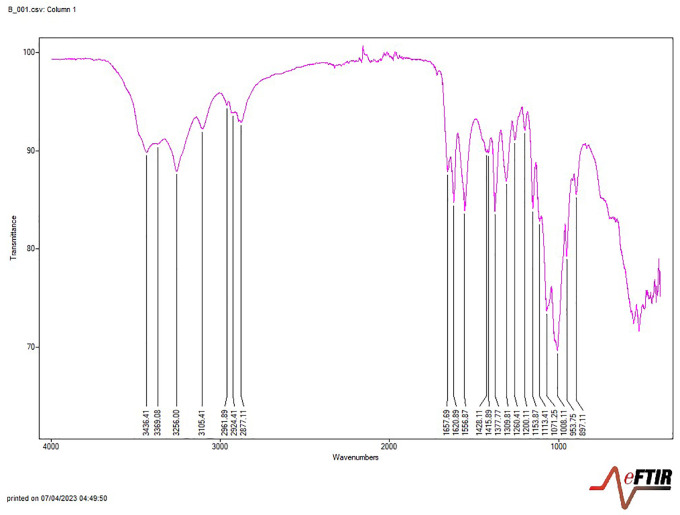
FTIR spectra of prawn chitosan.

The extraction of chitin yielded 17.78% from *Periplaneta americana* (cockroach) and 29.53% from *Macrobrachium vollenhovenii* (prawn), indicating a higher recovery from prawn exoskeletons (Table 3). Subsequent deacetylation produced chitosan yields of 11.56% and 28.13% from cockroach and prawn samples, respectively (Table 4), confirming prawn as the superior source in terms of extraction efficiency. Fourier-Transform Infrared (FTIR) spectroscopy revealed that chitosan obtained from both sources exhibited absorption bands consistent with characteristic functional groups of chitosan, including O–H, N–H, C–H, and C = O stretches. The observed spectra closely matched those of reference chitosan (Sigma Aldrich), with only minor shifts in band positions (Figs 1,2; Table 5), thereby validating the structural identity of the extracted polymers.

**Table 3:** Yield at each stage of chitin extraction and percentage chitin yield from the different extraction procedures (Values represent calculated percentages from experimental extraction).

Sample	Initial Weight (g)	Weight after Demineralization (g)	Weight after Deproteination (g)	Chitin Yield (%)
AC	9	–	1.60	17.78
ARP	15	6.81	4.43	29.53

**Table 4:** Overview of Chitosan Extraction Yields from Deacetylation of Chitin using the Different Procedures (Values represent calculated percentages from experimental extraction).

Sample	Initial Dry Weight of Sample (g)	Dry Weight of Chitin (g)	Dry Weight after Deacetylation (g)	Chitosan Yield (%)
AC	9	1.60	1.04	11.56
ARP	15	4.43	4.22	28.13

**Table 5:** A Comparison between the FTIR absorption wavelength of the extracted chitosan in the present study and a reference Chitosan from Sigma Aldrich.

Reference Chitosan [17]	Extracted AC Chitosan	Extracted ARP Chitosan	Functional Groups
Wave number (cm-1)	Wave number (cm-1)	Wave number (cm-1)	
3428	3360 − 3289	3436	(O-H) group (-NH2) group
2923	2916	2924	(CH2) in CH2OH group
2880	2876	2877	(C─H) in pyranose ring
1667−1623	1646	1657	(C = O) in the NHCOCH3 group (Amide I band)
–	1584	1556	(NH2) in NHCOCH3 group (amide II band)
1422	1421	1428	(CH2) in CH2OH group
1380	1375	1377	(CH3) in NHCOCH3 group
1322	1320	1309	(C–H) in pyranose ring
1155–1077	1149–1077	1159 − 1071	(C–O–C) (glycosidic linkage)
1031	1030	1023	(C–O) in primary OH group
897	893	897	Pyranose ring skeletal vibrations

The spectra show characteristic absorption peaks corresponding to functional groups of chitosan, confirming successful deacetylation. Key bands include O–H and N–H stretching vibrations, amide I, and amide II regions.

The spectra display characteristic functional group peaks associated with chitosan, indicating effective deacetylation. Prominent bands correspond to hydroxyl, amino, and amide groups.

Despite the higher yield obtained from prawn exoskeletons, cockroach-derived chitosan exhibited a greater degree of deacetylation (81.21%) compared to prawn-derived chitosan (68.79%) (Table 6).

**Table 6:** The Degree of Deacetylation (DD) of the chitosan obtained.

Sample	DD of Chitosan (%)
AC	81.21
ARP	68.79

**Fig 3:**
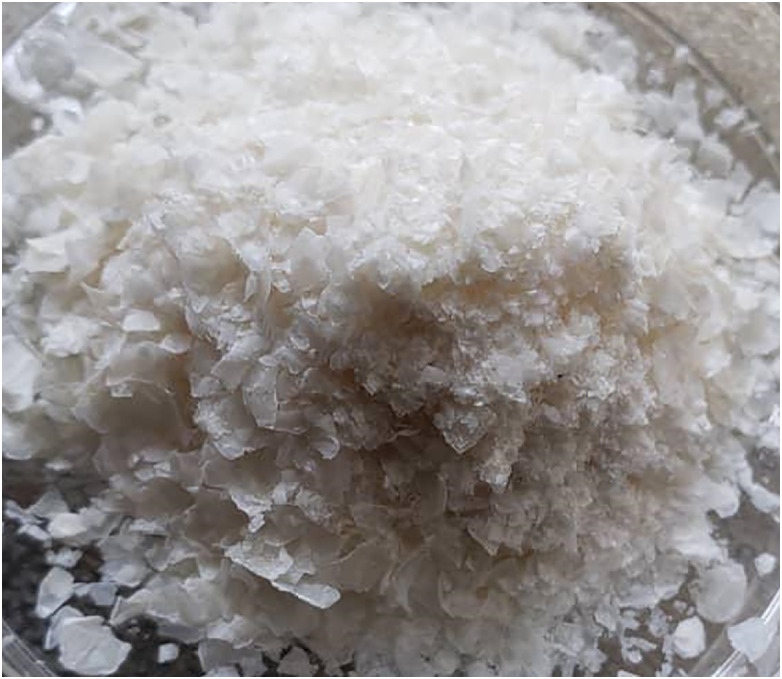
Chitosan obtained from prawn in the study.

**Fig 4:**
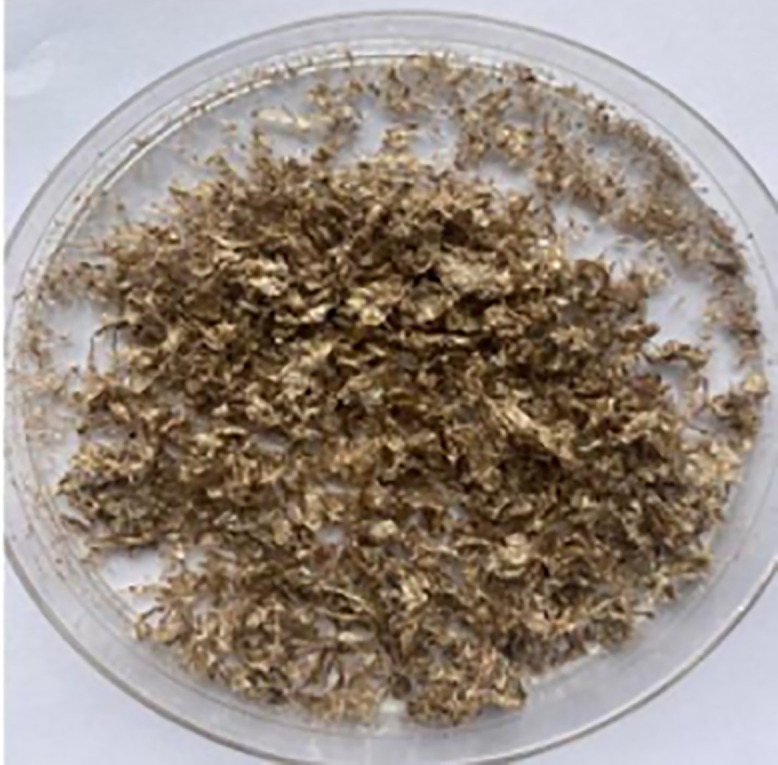
Chitosan obtained from cockroach in the study.

The extracted chitosan from both sources displayed distinct physical characteristics. Prawn derived chitosan appeared as a whitish, flaky solid with a relatively coarse particle consistency (Fig 3), while cockroach-derived chitosan exhibited a brown-hue coloration, flaky solid with brittle texture (Fig 4). These physical properties are consistent with purified chitosan reported in previous studies, reflecting successful deacetylation and removal of residual proteins and minerals.

The image shows the physical appearance of the extracted chitosan following deacetylation, highlighting its texture and yield characteristics

The image shows the final chitosan product after extraction and deacetylation, illustrating its physical properties and consistency.

## Discussion

The extraction and characterisation of chitosan from alternative biological sources remains a subject of growing interest due to the increasing demand for biodegradable and non-toxic biomaterials. Insects and crustaceans represent promising reservoirs of chitin and its derivative chitosan, with yields often influenced by species type, structural composition, and extraction methodology. In the present study, *Periplaneta americana* (cockroach) and *Macrobrachium vollenhovenii* (African river prawn) were evaluated as sources for chitosan production.

The yield of chitin from *P. americana* (17.18%)was higher than previous values for cockroach-derived chitin,which showed an average yield range of 12.17–15% [18,19]. Similarly, the chitin yield obtained from *M. vollenhovenii* (29.53%) exceeded many reported values for prawn and shrimp shells, with values ranging from 8.28-26.08% [15, 17, 20, 21]. Taken together, the yield observed in the present study may reflect the efficiency of the extraction process applied to the locally sourced species under the conditions tested.

The observed difference in chitin yield both from the prawn and cockroach from this study may also be attributed to environmental factors such as diet, humidity, temperature, and other ecological pressures that have been suggested affect exoskeleton thickness and rigidity. However, as these environmental variables were not directly measured or controlled in the present study, and given that such factors vary considerably across geographical regions, it is plausible that local developmental conditions could contribute to inter-study variability in chitin yield.. In this context, Nigeria, characterized by its relatively high humidity and temperature [22], could have provided favourable conditions that promoted the development of thicker, more rigid exoskeletons in cockroaches, potentially contributing to the higher chitin yield observed in our study. Consequently, geographical location and prevailing ecological factors should be considered when assessing the potential of arthropod populations as sources of chitin..

Chitosan, known for its unique properties, has garnered significant application in industries and medicines. Our study used a pressurized deacetylation method to extract chitosan from the exoskeleton of African River Prawn and Cockroach. We obtained a chitosan yield of 11.56% from cockroach chitin, higher than several reports using conventional deacetylation techniques, reporting a chitosan yield range of 7.28 - 7.4% [17, 19]. Also, we obtained a chitosan yield of 28.13% from the exoskeleton of African river prawn. This also exceeded those reported for *Macrobrachium rosenbergii* by Vupputuri et al [23] (25.99%) and from shrimp shell wastes by Mohanasrinivasan et al. [24] (17%) and Olafadehan et al. [17] (16.93%), indicating that reaction intensification can improve conversion efficiency within significantly reducing processing time (Table 7).

**Table 7:** Comparative overview of reported conventional deacetylation methods and the pressurized method used in this study.

Parameter	Conventional (Literature Range)	Pressurized (This Study)
Time	6 - 24 hrs	30 mins
DD	50 - 90%	68 - 81%
Yield	7 - 25%	11 - 28%

While pressurized deacetylation likely contributed to improved conversion efficiency, it is important to note that the variations in upstream conditions, including pre-treatment and demineralization durations, may also have influenced the final chitin and chitosan yields observed. The enhanced yield may be attributed to the pressurized deacetylation process, which likely increases the penetration of alkaline solution into the chitin matrix, and accelerates the removal of acetyl groups.. However, the absence of a conventional non-pressurised control extraction in this study may preclude direct attribution of yield improvement solely to the modified method. However, these findings suggest that the processing parameters including pressure, temperature, and reaction duration may play a critical role in maximising yield and quality, highlighting the potential of pressurized deacetylation for industrial-scale chitosan production.

The functional groups present in chitosan, such as hydroxyl (-OH), amino (-NH_2_), and acetyl (- COCH_3_) groups, contribute to its wide biological applicability, including interactions with cell membranes, proteins, and DNA, as well as roles in drug delivery, surface modification, and bioconjugation [25]. The FTIR spectroscopy confirmed the successful conversion of chitin to chitosan in both biological sources.

The spectra of chitosan extracted from the cockroach sample showed an absorption band at 3360–3289 cm-1, indicating the presence of OH and NH2 groups. The major amide bands were observed at 1646 cm-1 (carbonyl, Amide I), 1584 cm-1 (NH2, amide II band), and 1320 cm-1 (amide III in C-N). The characteristic vibrations of C-H in chitosan were evident at 1421 cm-1 and 1375 cm-1. The oxygen stretching of the glycosidic linkage was observed at 1149–1077 cm-1. In addition, in the FTIR spectra of chitosan extracted from the prawn sample, the chitosan exhibited stretching vibration of the OH group at 3432 cm^-1^. The absorption band at 1657 cm^-1^ indicated the presence of the C = O (carbonyl, Amide I) group. Similarly, the presence of the amide II band (N-H bending) was indicated by the absorption band at 1557 cm^-1^. The -CH2 groups were observed at 2918 cm^-1^ and 1423 cm^-1^. The oxygen stretching of the glycosidic linkage was observed at 1154 cm^-1^. The spectra of chitosan extracted from cockroach samples showed close similarities to previous reports [26,27]. Likewise, the FTIR spectra of chitosan extracted from prawn samples exhibited absorption bands comparable to reports of Kjartansson et al. and Balkhande and Ratnakar [19 20].

The observed differences in peak intensity between cockroach and prawn-derived chitosan suggest source-dependent variation in deacetylation behaviour. The more pronounced reduction of amide-associated bands observed in cockroach-derived chitosan aligns with its higher degree of deacetylation. These differences may reflect variations in chitin crystallinity, acetyl group accessibility, and diffusion kinetics under short-duration, high-temperature alkaline treatment, an insight into how biological origin interacts with processing conditions to influence deacetylation efficiency.

Importantly,the degree of deacetylation (DD), which quantifies the conversion of N-acetyl-D-glucosamine units, is an important indicator of chitosan physicochemical properties, solubility, and bioactivity, making it a key determinant of functional applicability [28,29]. In the present study, cockroach-derived chitosan exhibited a DD of 81.21%, consistent with effective acetyl group removal under pressurised alkaline conditions. This exceeds the 70% reported by [30] but lower than the 90.43% documented by [18], likely reflecting the differences in deacetylation duration and processing conditions, which prioritises rapid conversion rather than maximal deacetylation. In contrast prawn-derived chitosan, exhibited a DD value of 68.79%,lower than the 74.82% reported by Mohanasrinivasan et al. [24] and the 70–85% range reported by Ahing and Wid [31] for shrimp shells in Sabah, using conventional deacetylation. Isa et al. [32] reported a DD value of 50.64%, while Olafadehan et al. [17] achieved a markedly higher value of 89.73% from local shrimp sources in Nigeria. These differences may be attributable to the higher mineral content and structural rigidity in the source of chitin (species, habitat, and environmental conditions) which can limit alkali penetration during the short-duration treatment [33]. However, the observed DD in both chitosan sources suggests the efficiency of pressurized deacetylation in producing chitosan with a substantial conversion of N-acetyl-D-glucosamine to D-glucosamine units. However, the lower value observed for the prawn suggests that the short intense, high-concentration alkaline conditions may promote partial rather than uniform deacetylation in the crustacean chitin, resulting in reduced overall DD despite high chitosan yield.

Despite exhibiting a higher DD, cockroach-derived chitosan displayed a brown-hued coloration, whereas prawn-derived chitosan appeared lighter and more uniform. This observation indicates that DD alone is insufficient to fully describe the obtained chitosan quality, as residual pigments persist following deproteinization, highlighting the need for complementary purity assessments. Furthermore, the absence of a parallel conventional non-pressurised deacetylation control performed under identical experimental conditions is another limitation of this study. Although our results compare favorably with yields and DD values reported in literature using conventional 6–24-hour methods, a direct side-by-side experimental comparison would provide stronger evidence of performance enhancement. It is important to note that this study was designed as an exploratory feasibility investigation aimed at establishing proof-of-concept for pressurized deacetylation using locally sourced arthropod exoskeletons. Consequently, no inferential statistical analyses were performed, and results are presented descriptively. Further studies will incorporate a controlled comparison between pressurised and traditional alkaline deacetylation approaches to quantitatively assess differences in yield, degree of deacetylation, reaction efficiency and processing time.

Overall, the results demonstrate that pressurised alkaline deacetylation enables rapid conversion of chitin to chitosan with moderate-to-high DD and competitive yields from both insect and crustacean sources. However, differences in upstream processing conditions, lack of replicate extractions, and absence of a conventional deacetylation control limit direct performance comparisons. Furthermore, the potential of *P. americana* as established in this study should however be viewed within alternative sustainability frameworks. Cockroaches possess rapid reproductive cycles, high biomass conversion efficiency and ability to thrive on organic wastes, making controlled insect farming a conceivable approach for biomass generation. Additionally, in regions lacking large-scale seafood processing infrastructure, insect-based chitin sources may represent a decentralized and locally adaptable alternative. Therefore, while cockroach-derived chitosan may not immediately replace crustacean waste streams for industrial-scale production, it presents a complementary and potential scalable source under circular bioeconomy models.

In addition, the method described in this study has been formally patented (Patent File No: F/PT/NC/2024/12757) under the title *“Chitosan Extraction Method Using Cockroach (Periplaneta americana) Exoskeleton”*. This patent highlights the novelty of the pressurized deacetylation approach and underscores its potential for industrial-scale application in biotechnology and biomedicine.

## Conclusion

This study demonstrates the feasibility of extracting chitosan from locally-sourced West African river prawns (*Macrobrachium vollenhovenii*) and American cockroaches (*Periplaneta americana*) using a modified pressurised alkaline deacetylation approach. Our findings demonstrate that this method enabled rapid conversion of chitin to chitosan within a substantially reduced processing time compared to conventional deacetylation protocols, yielding chitosan with moderate-to-high degrees of deacetylation. Under the conditions tested, cockroach-derived chitosan exhibited a DD of 81.21%, while prawn-derived chitosan exhibited a DD of 68.79%, confirming effective acetyl group removal as supported by FTIR analysis. Taken together, the results highlight the potential of pressurized alkaline treatment as a promising strategy for accelerating chitosan production from both insect and crustacean sources. Future studies incorporating standardized pre-treatment protocols, replicated experiments, direct comparison with conventional deacetylation methods, and comprehensive physicochemical characterization such as X-ray diffraction (XRD), scanning electron microscopy (SEM), crystallinity index determination, and molecular weight analysis will be essential to fully assess yield optimization, product quality, and provide deeper insights into structural organizations, surface morphology and polymer properties, and scalability.

## Supporting information

S1 File Supplementary data containing cockroach and prawn FTIR spectra values, Chitin yield, Chitosan yield, degree of deacetylation analyses, and Values of chitin and chitosan extraction results from both sources. (ZIP)

## References

[1] Aibani N, Rai R, Patel P, Cuddihy G, Wasan EK. Chitosan Nanoparticles at the Biological Interface: Implications for Drug Delivery. Pharmaceutics. 2021;13(10):1686. doi: 10.3390/pharmaceutics13101686 34683979 PMC8540112

[2] Jiménez‐Gómez CP, Cecilia JA. Chitosan: A natural biopolymer with a wide and varied range of applications. Molecules. 2020;25. doi: 10.3390/molecules25173981 PMC7504732 32882899

[3] Kaur S, Dhillon GS. The versatile biopolymer chitosan: potential sources, evaluation of extraction methods and applications. Crit Rev Microbiol. 2014;40(2):155–75. doi: 10.3109/1040841X.2013.770385 23488873

[4] Vieira H, Lestre GM, Solstad RG, Cabral AE, Botelho A, Helbig C, et al. Current and Expected Trends for the Marine Chitin/Chitosan and Collagen Value Chains. Mar Drugs. 2023;21(12):605. doi: 10.3390/md21120605 38132926 PMC10744996

[5] Harugade A, Sherje AP, Pethe A. Chitosan: A review on properties, biological activities and recent progress in biomedical applications. Reactive and Functional Polymers. 2023;191:105634. doi: 10.1016/j.reactfunctpolym.2023.105634

[6] Shariatinia Z. Pharmaceutical applications of chitosan. Adv Colloid Interface Sci. 2019;263:131–94. doi: 10.1016/j.cis.2018.11.008 30530176

[7] Thambiliyagodage C, Jayanetti M, Mendis A, Ekanayake G, Liyanaarachchi H, Vigneswaran S. Recent Advances in Chitosan-Based Applications-A Review. Materials (Basel). 2023;16(5):2073. doi: 10.3390/ma16052073 36903188 PMC10004736

[8] Mersmann L, Souza VGL, Fernando AL. Green Processes for Chitin and Chitosan Production from Insects: Current State, Challenges, and Opportunities. Polymers (Basel). 2025;17(9):1185. doi: 10.3390/polym17091185 40362968 PMC12073625

[9] Yi K, Miao S, Yang B, Li S, Lu Y. Harnessing the potential of chitosan and its derivatives for enhanced functionalities in food applications. Foods. 2024;13. doi: 10.3390/foods13030439 PMC10855628 38338575

[10] Pellis A, Guebitz GM, Nyanhongo GS. Chitosan: Sources, Processing and Modification Techniques. Gels. 2022;8(7):393. doi: 10.3390/gels8070393 35877478 PMC9322947

[11] Ma J, Faqir Y, Tan C, Khaliq G. Terrestrial insects as a promising source of chitosan and recent developments in its application for various industries. Food Chem. 2022;373(Pt A):131407. doi: 10.1016/j.foodchem.2021.131407 34715633

[12] Liyanage CS, Gonapinuwala ST, Fernando CAN, de Croos MDST. A Simple and Effective Method to Extract Chitosan from Crustacean Shell Waste. Journal of Aquatic Food Product Technology. 2023;32(4):396–415. doi: 10.1080/10498850.2023.2228793

[13] Saenz-Mendoza AI, Zamudio-Flores PB, García-Anaya MC, Velasco CR, Acosta-Muñiz CH, Espino-Díaz M, et al. Insects as a potential source of chitin and chitosan: Physicochemical, morphological and structural characterization. -A review. Emir J Food Agric. 2023. doi: 10.9755/ejfa.2023.v35.i5.3095

[14] Okrikata E, Yusuf AO. A study on the diversity and relative abundance of insect fauna in Wukari, Taraba State, Nigeria. International Journal of Advanced Biological and Biomedical Research. 2019. doi: 10.33945/sami/ijabbr.2019.2.4

[15] Sagheer FAA, Al-Sughayer MA, Muslim S, Elsabee MZ. Extraction and characterization of chitin and chitosan from marine sources in Arabian Gulf. Carbohydrate Polymers. 2009;77(2):410–9. doi: 10.1016/j.carbpol.2009.01.032

[16] Sabnis S, Block LH. Improved infrared spectroscopic method for the analysis of degree of N-deacetylation of chitosan. Polymer Bulletin. 1997;39(1):67–71. doi: 10.1007/s002890050121

[17] Olaosebikan AO, Kehinde OA, Tolulase OA, Victor EB. Extraction and characterization of chitin and chitosan from Callinectes amnicola and Penaeus notialis shell wastes. J Chem Eng Mater Sci. 2021;12(1):1–30. doi: 10.5897/jcems2020.0353

[18] Kim M-W, Song Y-S, Seo D-J, Han YS, Jo YH, Noh MY, et al. Extraction of Chitin and Chitosan from the Exoskeleton of the Cockroach (Periplaneta americana L.). J Chitin Chitosan. 2017;22(2):76–81. doi: 10.17642/jcc.22.2.2

[19] Basseri H, Bakhtiyari R, Hashemi SJ, Baniardelani M, Shahraki H, Hosainpour L. Antibacterial/Antifungal Activity of Extracted Chitosan From American Cockroach (Dictyoptera: Blattidae) and German Cockroach (Blattodea: Blattellidae). J Med Entomol. 2019;56(5):1208–14. doi: 10.1093/jme/tjz082 31139829

[20] Kjartansson GT, Zivanovic S, Kristbergsson K, Weiss J. Sonication-assisted extraction of chitin from shells of fresh water prawns (Macrobrachium rosenbergii). J Agric Food Chem. 2006;54(9):3317–23. doi: 10.1021/jf052184c 16637691

[21] Balkhande J, Ratnakar P. Extraction And Ftir Analysis Of Chitosan From Freshwater Crab Barytelphusa Cunicularis And Freshwater Prawn Macrobrachium Rosenbergii. 2019:370–374.

[22] Dorcas Mobolade T, Pourvahidi P. Bioclimatic Approach for Climate Classification of Nigeria. Sustainability. 2020;12(10):4192. doi: 10.3390/su12104192

[23] Panchakshari V, Srikanth K, Krishna PV, Babu ChS. Extraction of Chitin and Chitosan from Biowaste of Scampi Macrobrichum rosenbergii and Tiger Shrimp Penaeus monodon. IntJCurrMicrobiolAppSci. 2016;5(7):751–8. doi: 10.20546/ijcmas.2016.507.086

[24] Mohanasrinivasan V, Mishra M, Paliwal J, Singh S, Selvarajan E, Suganthi V, et al. Studies on heavy metal removal efficiency and antibacterial activity of chitosan prepared from shrimp shell waste. 3 Biotech. 2013. doi: 10.1007/s13205-013-0140-6 PMC3964254 28324448

[25] Dash M, Chiellini F, Ottenbrite RM, Chiellini E. Chitosan—A versatile semi-synthetic polymer in biomedical applications. Progress in Polymer Science. 2011;36(8):981–1014. doi: 10.1016/j.progpolymsci.2011.02.001

[26] Umar ZG, Abalaka ME, Daniyan SY, Babayi H, Adeniyi KA. Physicochemical and bio-metabolite characterizations of chitosan isolated from American cockroach (Periplaneta americana) and cricket (Acheta domesticus). BIOMED nat appl sci. 2022;02(01):25–36. doi: 10.53858/bnas02012536

[27] Wanule D. Extraction and FTIR Analysis of Chitosan from American Cockroach, Periplaneta americana. 2014.

[28] Novikov VY, Derkach SR, Konovalova IN, Dolgopyatova NV, Kuchina YA. Mechanism of Heterogeneous Alkaline Deacetylation of Chitin: A Review. Polymers (Basel). 2023;15(7):1729. doi: 10.3390/polym15071729 37050343 PMC10097213

[29] Aranaz I, Alcántara AR, Civera MC, Arias C, Elorza B, Heras Caballero A, et al. Chitosan: An Overview of Its Properties and Applications. Polymers (Basel). 2021;13(19):3256. doi: 10.3390/polym13193256 34641071 PMC8512059

[30] Badawy R, Mohamed H. Chitin extraction, composition of different six insect species and their comparable characteristics with that of the shrimp. Journal of American Science. 2015;11:127.

[31] Román-Doval R, Torres-Arellanes SP, Tenorio-Barajas AY, Gómez-Sánchez A, Valencia-Lazcano AA. Chitosan: Properties and Its Application in Agriculture in Context of Molecular Weight. Polymers (Basel). 2023;15(13):2867. doi: 10.3390/polym15132867 37447512 PMC10346603

[32] Isa M, Ameh A, Tijjani M, Adama K. Extraction and characterization of chitin and chitosan from Nigerian shrimps. Int J Bio Chem Sci. 2012;6(1). doi: 10.4314/ijbcs.v6i1.40

[33] Sánchez-Machado DI, López-Cervantes J, Escárcega-Galaz AA, Campas-Baypoli ON, Martínez-Ibarra DM, Rascón-León S. Measurement of the degree of deacetylation in chitosan films by FTIR, 1H NMR and UV spectrophotometry. MethodsX. 2024;12:102583. doi: 10.1016/j.mex.2024.102583 38313694 PMC10837090

