# Supplementary material for: Biotechnology-driven extraction and characterisation of Chitosan from the West African river prawn (Macrobrachium vollenhovenii) and American Cockroach (Periplaneta americana) using a modified approach
Source: PLoS One. 2026 May 28;21(5):e0349133. doi: 10.1371/journal.pone.0349133 (PMC13218523; doi:10.1371/journal.pone.0349133)
Supplement: S1 File — (ZIP) [file pone.0349133.s001.zip › CockroachSpectraValues.pdf]

Created as	Sample 00:	June 20 2023
cm-1	%T	
4000	99.46	
3999	99.45	
3998	99.45	
3997	99.44	
3996	99.44	
3995	99.43	
3994	99.43	
3993	99.42	
3992	99.42	
3991	99.42	
3990	99.42	
3989	99.43	
3988	99.43	
3987	99.44	
3986	99.45	
3985	99.46	
3984	99.46	
3983	99.45	
3982	99.44	
3981	99.44	
3980	99.44	
3979	99.45	
3978	99.45	
3977	99.45	
3976	99.44	
3975	99.44	
3974	99.44	
3973	99.45	
3972	99.45	
3971	99.46	
3970	99.46	
3969	99.45	

3968	99.45
3967	99.44
3966	99.43
3965	99.43
3964	99.43
3963	99.44
3962	99.44
3961	99.44
3960	99.44
3959	99.44
3958	99.44
3957	99.45
3956	99.45
3955	99.45
3954	99.45
3953	99.44
3952	99.44
3951	99.43
3950	99.43
3949	99.43
3948	99.43
3947	99.43
3946	99.43
3945	99.44
3944	99.45
3943	99.46
3942	99.46
3941	99.46
3940	99.45
3939	99.45
3938	99.45
3937	99.45
3936	99.44
3935	99.43

3934	99.42
3933	99.42
3932	99.43
3931	99.45
3930	99.47
3929	99.48
3928	99.48
3927	99.47
3926	99.46
3925	99.45
3924	99.45
3923	99.45
3922	99.45
3921	99.45
3920	99.45
3919	99.46
3918	99.46
3917	99.46
3916	99.46
3915	99.46
3914	99.46
3913	99.46
3912	99.45
3911	99.45
3910	99.45
3909	99.45
3908	99.45
3907	99.44
3906	99.43
3905	99.41
3904	99.4
3903	99.39
3902	99.41
3901	99.43

3900	99.45
3899	99.46
3898	99.46
3897	99.45
3896	99.44
3895	99.44
3894	99.45
3893	99.46
3892	99.47
3891	99.47
3890	99.47
3889	99.47
3888	99.47
3887	99.47
3886	99.47
3885	99.46
3884	99.45
3883	99.46
3882	99.48
3881	99.5
3880	99.5
3879	99.48
3878	99.46
3877	99.45
3876	99.45
3875	99.45
3874	99.45
3873	99.44
3872	99.43
3871	99.43
3870	99.43
3869	99.43
3868	99.43
3867	99.44

3866	99.46
3865	99.48
3864	99.49
3863	99.49
3862	99.49
3861	99.49
3860	99.48
3859	99.48
3858	99.48
3857	99.48
3856	99.47
3855	99.46
3854	99.46
3853	99.46
3852	99.46
3851	99.46
3850	99.46
3849	99.46
3848	99.46
3847	99.46
3846	99.47
3845	99.49
3844	99.51
3843	99.51
3842	99.51
3841	99.5
3840	99.48
3839	99.48
3838	99.49
3837	99.5
3836	99.5
3835	99.5
3834	99.5
3833	99.5

3832	99.5
3831	99.5
3830	99.5
3829	99.51
3828	99.51
3827	99.51
3826	99.5
3825	99.49
3824	99.47
3823	99.46
3822	99.47
3821	99.47
3820	99.48
3819	99.49
3818	99.5
3817	99.51
3816	99.52
3815	99.52
3814	99.52
3813	99.51
3812	99.51
3811	99.5
3810	99.49
3809	99.47
3808	99.47
3807	99.46
3806	99.45
3805	99.45
3804	99.46
3803	99.48
3802	99.49
3801	99.49
3800	99.48
3799	99.48

3798	99.49
3797	99.5
3796	99.51
3795	99.51
3794	99.51
3793	99.51
3792	99.51
3791	99.5
3790	99.5
3789	99.49
3788	99.49
3787	99.49
3786	99.49
3785	99.49
3784	99.49
3783	99.48
3782	99.47
3781	99.48
3780	99.49
3779	99.51
3778	99.51
3777	99.51
3776	99.5
3775	99.5
3774	99.5
3773	99.5
3772	99.49
3771	99.49
3770	99.49
3769	99.48
3768	99.47
3767	99.47
3766	99.47
3765	99.46

3764	99.45
3763	99.44
3762	99.45
3761	99.46
3760	99.48
3759	99.49
3758	99.49
3757	99.49
3756	99.48
3755	99.48
3754	99.47
3753	99.45
3752	99.42
3751	99.39
3750	99.37
3749	99.37
3748	99.38
3747	99.4
3746	99.41
3745	99.4
3744	99.38
3743	99.36
3742	99.35
3741	99.36
3740	99.37
3739	99.38
3738	99.39
3737	99.41
3736	99.45
3735	99.5
3734	99.52
3733	99.52
3732	99.5
3731	99.48

3730	99.47
3729	99.45
3728	99.44
3727	99.42
3726	99.4
3725	99.4
3724	99.4
3723	99.41
3722	99.43
3721	99.45
3720	99.46
3719	99.47
3718	99.48
3717	99.48
3716	99.48
3715	99.48
3714	99.47
3713	99.46
3712	99.46
3711	99.46
3710	99.46
3709	99.47
3708	99.47
3707	99.47
3706	99.46
3705	99.45
3704	99.44
3703	99.43
3702	99.43
3701	99.42
3700	99.42
3699	99.41
3698	99.41
3697	99.41

3696	99.4
3695	99.4
3694	99.4
3693	99.41
3692	99.43
3691	99.45
3690	99.47
3689	99.47
3688	99.45
3687	99.42
3686	99.41
3685	99.41
3684	99.42
3683	99.42
3682	99.43
3681	99.42
3680	99.41
3679	99.4
3678	99.37
3677	99.36
3676	99.35
3675	99.34
3674	99.32
3673	99.3
3672	99.3
3671	99.32
3670	99.34
3669	99.34
3668	99.34
3667	99.33
3666	99.33
3665	99.32
3664	99.32
3663	99.32

3662	99.31
3661	99.31
3660	99.3
3659	99.28
3658	99.27
3657	99.26
3656	99.25
3655	99.24
3654	99.23
3653	99.22
3652	99.21
3651	99.18
3650	99.16
3649	99.15
3648	99.15
3647	99.16
3646	99.15
3645	99.14
3644	99.13
3643	99.11
3642	99.1
3641	99.08
3640	99.06
3639	99.04
3638	99.01
3637	98.99
3636	98.98
3635	98.97
3634	98.96
3633	98.94
3632	98.92
3631	98.89
3630	98.86
3629	98.85

3628	98.84
3627	98.84
3626	98.83
3625	98.83
3624	98.82
3623	98.8
3622	98.78
3621	98.76
3620	98.74
3619	98.71
3618	98.68
3617	98.65
3616	98.63
3615	98.63
3614	98.63
3613	98.63
3612	98.61
3611	98.59
3610	98.58
3609	98.57
3608	98.55
3607	98.53
3606	98.51
3605	98.48
3604	98.45
3603	98.43
3602	98.41
3601	98.4
3600	98.39
3599	98.38
3598	98.36
3597	98.34
3596	98.32
3595	98.3

3594	98.28
3593	98.26
3592	98.24
3591	98.22
3590	98.18
3589	98.14
3588	98.1
3587	98.07
3586	98.06
3585	98.04
3584	98.03
3583	98.02
3582	98.01
3581	97.98
3580	97.96
3579	97.93
3578	97.9
3577	97.88
3576	97.85
3575	97.83
3574	97.8
3573	97.77
3572	97.75
3571	97.73
3570	97.72
3569	97.71
3568	97.69
3567	97.66
3566	97.62
3565	97.58
3564	97.55
3563	97.51
3562	97.48
3561	97.44

3560	97.41
3559	97.38
3558	97.34
3557	97.3
3556	97.26
3555	97.23
3554	97.2
3553	97.18
3552	97.16
3551	97.14
3550	97.12
3549	97.09
3548	97.06
3547	97.03
3546	97
3545	96.99
3544	96.98
3543	96.97
3542	96.97
3541	96.95
3540	96.92
3539	96.88
3538	96.85
3537	96.82
3536	96.8
3535	96.79
3534	96.78
3533	96.76
3532	96.74
3531	96.7
3530	96.67
3529	96.64
3528	96.61
3527	96.58

3526	96.55
3525	96.51
3524	96.47
3523	96.43
3522	96.4
3521	96.37
3520	96.35
3519	96.33
3518	96.31
3517	96.28
3516	96.25
3515	96.22
3514	96.2
3513	96.17
3512	96.13
3511	96.1
3510	96.06
3509	96.03
3508	96
3507	95.97
3506	95.95
3505	95.91
3504	95.88
3503	95.84
3502	95.81
3501	95.77
3500	95.74
3499	95.71
3498	95.67
3497	95.64
3496	95.6
3495	95.56
3494	95.52
3493	95.49

3492	95.45
3491	95.42
3490	95.38
3489	95.34
3488	95.3
3487	95.26
3486	95.23
3485	95.2
3484	95.16
3483	95.12
3482	95.08
3481	95.03
3480	94.99
3479	94.96
3478	94.94
3477	94.92
3476	94.9
3475	94.86
3474	94.82
3473	94.78
3472	94.73
3471	94.69
3470	94.65
3469	94.62
3468	94.58
3467	94.56
3466	94.53
3465	94.49
3464	94.46
3463	94.43
3462	94.4
3461	94.37
3460	94.35
3459	94.32

3458	94.29
3457	94.27
3456	94.24
3455	94.22
3454	94.19
3453	94.16
3452	94.12
3451	94.09
3450	94.06
3449	94.04
3448	94.01
3447	93.98
3446	93.96
3445	93.93
3444	93.92
3443	93.89
3442	93.87
3441	93.84
3440	93.82
3439	93.8
3438	93.79
3437	93.79
3436	93.79
3435	93.79
3434	93.78
3433	93.76
3432	93.74
3431	93.71
3430	93.69
3429	93.67
3428	93.66
3427	93.66
3426	93.67
3425	93.67

3424	93.67
3423	93.67
3422	93.66
3421	93.65
3420	93.64
3419	93.62
3418	93.62
3417	93.61
3416	93.61
3415	93.6
3414	93.59
3413	93.57
3412	93.55
3411	93.55
3410	93.55
3409	93.55
3408	93.55
3407	93.55
3406	93.55
3405	93.54
3404	93.53
3403	93.52
3402	93.52
3401	93.51
3400	93.5
3399	93.48
3398	93.46
3397	93.43
3396	93.41
3395	93.4
3394	93.39
3393	93.37
3392	93.35
3391	93.33

3390	93.31
3389	93.31
3388	93.31
3387	93.31
3386	93.29
3385	93.27
3384	93.23
3383	93.2
3382	93.18
3381	93.17
3380	93.17
3379	93.16
3378	93.16
3377	93.15
3376	93.14
3375	93.13
3374	93.11
3373	93.1
3372	93.08
3371	93.05
3370	93.02
3369	92.98
3368	92.96
3367	92.94
3366	92.93
3365	92.93
3364	92.91
3363	92.9
3362	92.88
3361	92.87
3360	92.88
3359	92.89
3358	92.91
3357	92.92

3356	92.93
3355	92.94
3354	92.94
3353	92.95
3352	92.96
3351	92.97
3350	92.98
3349	92.99
3348	93
3347	93.01
3346	93
3345	93
3344	93
3343	93.01
3342	93.01
3341	93.03
3340	93.04
3339	93.06
3338	93.08
3337	93.09
3336	93.1
3335	93.11
3334	93.11
3333	93.12
3332	93.12
3331	93.12
3330	93.12
3329	93.12
3328	93.12
3327	93.11
3326	93.11
3325	93.11
3324	93.11
3323	93.12

3322	93.12
3321	93.11
3320	93.1
3319	93.1
3318	93.08
3317	93.07
3316	93.06
3315	93.04
3314	93.02
3313	93.01
3312	92.99
3311	92.98
3310	92.97
3309	92.96
3308	92.95
3307	92.95
3306	92.94
3305	92.94
3304	92.93
3303	92.94
3302	92.94
3301	92.94
3300	92.93
3299	92.93
3298	92.93
3297	92.94
3296	92.94
3295	92.94
3294	92.93
3293	92.91
3292	92.9
3291	92.89
3290	92.89
3289	92.89

3288	92.9
3287	92.9
3286	92.91
3285	92.92
3284	92.93
3283	92.94
3282	92.96
3281	92.98
3280	93
3279	93.01
3278	93.02
3277	93.02
3276	93.02
3275	93.02
3274	93.02
3273	93.02
3272	93.03
3271	93.03
3270	93.04
3269	93.06
3268	93.07
3267	93.09
3266	93.1
3265	93.11
3264	93.12
3263	93.14
3262	93.16
3261	93.17
3260	93.19
3259	93.2
3258	93.21
3257	93.22
3256	93.23
3255	93.23

3254	93.23
3253	93.24
3252	93.25
3251	93.26
3250	93.27
3249	93.28
3248	93.29
3247	93.29
3246	93.29
3245	93.29
3244	93.29
3243	93.31
3242	93.32
3241	93.33
3240	93.34
3239	93.35
3238	93.36
3237	93.37
3236	93.38
3235	93.39
3234	93.4
3233	93.4
3232	93.4
3231	93.4
3230	93.41
3229	93.42
3228	93.42
3227	93.43
3226	93.43
3225	93.43
3224	93.44
3223	93.46
3222	93.48
3221	93.49

3220	93.5
3219	93.5
3218	93.5
3217	93.51
3216	93.52
3215	93.53
3214	93.53
3213	93.53
3212	93.53
3211	93.53
3210	93.53
3209	93.53
3208	93.54
3207	93.55
3206	93.57
3205	93.58
3204	93.6
3203	93.6
3202	93.6
3201	93.61
3200	93.61
3199	93.62
3198	93.63
3197	93.65
3196	93.67
3195	93.68
3194	93.7
3193	93.71
3192	93.72
3191	93.73
3190	93.74
3189	93.74
3188	93.75
3187	93.76

3186	93.77
3185	93.77
3184	93.77
3183	93.78
3182	93.8
3181	93.82
3180	93.84
3179	93.85
3178	93.86
3177	93.87
3176	93.88
3175	93.88
3174	93.89
3173	93.9
3172	93.91
3171	93.93
3170	93.96
3169	93.97
3168	93.99
3167	94
3166	94
3165	94.02
3164	94.03
3163	94.04
3162	94.05
3161	94.05
3160	94.06
3159	94.07
3158	94.09
3157	94.12
3156	94.14
3155	94.16
3154	94.17
3153	94.18

3152	94.19
3151	94.19
3150	94.2
3149	94.22
3148	94.24
3147	94.26
3146	94.28
3145	94.29
3144	94.3
3143	94.31
3142	94.33
3141	94.35
3140	94.36
3139	94.38
3138	94.39
3137	94.41
3136	94.43
3135	94.45
3134	94.47
3133	94.48
3132	94.48
3131	94.48
3130	94.49
3129	94.49
3128	94.5
3127	94.51
3126	94.52
3125	94.53
3124	94.54
3123	94.56
3122	94.58
3121	94.61
3120	94.62
3119	94.63

3118	94.63
3117	94.64
3116	94.64
3115	94.65
3114	94.65
3113	94.65
3112	94.65
3111	94.66
3110	94.67
3109	94.68
3108	94.7
3107	94.71
3106	94.72
3105	94.73
3104	94.74
3103	94.75
3102	94.76
3101	94.77
3100	94.78
3099	94.79
3098	94.8
3097	94.82
3096	94.83
3095	94.83
3094	94.84
3093	94.84
3092	94.86
3091	94.88
3090	94.89
3089	94.91
3088	94.93
3087	94.94
3086	94.95
3085	94.96

3084	94.97
3083	94.98
3082	95
3081	95.02
3080	95.04
3079	95.05
3078	95.07
3077	95.08
3076	95.1
3075	95.11
3074	95.13
3073	95.15
3072	95.17
3071	95.19
3070	95.21
3069	95.22
3068	95.22
3067	95.23
3066	95.24
3065	95.25
3064	95.27
3063	95.3
3062	95.33
3061	95.36
3060	95.39
3059	95.41
3058	95.43
3057	95.44
3056	95.45
3055	95.47
3054	95.48
3053	95.49
3052	95.51
3051	95.53

3050	95.55
3049	95.56
3048	95.57
3047	95.58
3046	95.59
3045	95.61
3044	95.64
3043	95.67
3042	95.7
3041	95.73
3040	95.74
3039	95.75
3038	95.76
3037	95.77
3036	95.79
3035	95.81
3034	95.82
3033	95.83
3032	95.85
3031	95.86
3030	95.87
3029	95.89
3028	95.9
3027	95.92
3026	95.93
3025	95.94
3024	95.95
3023	95.97
3022	95.99
3021	96
3020	96.01
3019	96.02
3018	96.03
3017	96.04

3016	96.05
3015	96.05
3014	96.05
3013	96.06
3012	96.07
3011	96.1
3010	96.12
3009	96.14
3008	96.16
3007	96.17
3006	96.18
3005	96.19
3004	96.19
3003	96.19
3002	96.19
3001	96.18
3000	96.18
2999	96.19
2998	96.2
2997	96.21
2996	96.22
2995	96.22
2994	96.23
2993	96.23
2992	96.24
2991	96.23
2990	96.23
2989	96.21
2988	96.19
2987	96.17
2986	96.15
2985	96.15
2984	96.15
2983	96.17

2982	96.19
2981	96.21
2980	96.21
2979	96.2
2978	96.17
2977	96.15
2976	96.14
2975	96.13
2974	96.12
2973	96.12
2972	96.12
2971	96.12
2970	96.11
2969	96.09
2968	96.07
2967	96.03
2966	96.01
2965	95.98
2964	95.97
2963	95.96
2962	95.96
2961	95.95
2960	95.93
2959	95.92
2958	95.9
2957	95.9
2956	95.89
2955	95.89
2954	95.88
2953	95.86
2952	95.85
2951	95.83
2950	95.8
2949	95.78

2948	95.75
2947	95.73
2946	95.71
2945	95.68
2944	95.65
2943	95.61
2942	95.57
2941	95.53
2940	95.48
2939	95.43
2938	95.39
2937	95.36
2936	95.33
2935	95.3
2934	95.27
2933	95.23
2932	95.19
2931	95.15
2930	95.11
2929	95.08
2928	95.04
2927	95
2926	94.97
2925	94.93
2924	94.91
2923	94.89
2922	94.89
2921	94.88
2920	94.87
2919	94.85
2918	94.84
2917	94.83
2916	94.84
2915	94.86

2914	94.87
2913	94.87
2912	94.86
2911	94.84
2910	94.82
2909	94.8
2908	94.79
2907	94.79
2906	94.78
2905	94.78
2904	94.78
2903	94.77
2902	94.77
2901	94.77
2900	94.76
2899	94.75
2898	94.73
2897	94.71
2896	94.69
2895	94.67
2894	94.66
2893	94.65
2892	94.63
2891	94.61
2890	94.6
2889	94.59
2888	94.57
2887	94.56
2886	94.54
2885	94.52
2884	94.5
2883	94.48
2882	94.47
2881	94.46

2880	94.45
2879	94.43
2878	94.41
2877	94.4
2876	94.4
2875	94.41
2874	94.42
2873	94.44
2872	94.45
2871	94.47
2870	94.49
2869	94.51
2868	94.53
2867	94.55
2866	94.57
2865	94.6
2864	94.63
2863	94.66
2862	94.68
2861	94.7
2860	94.72
2859	94.74
2858	94.76
2857	94.78
2856	94.8
2855	94.82
2854	94.84
2853	94.88
2852	94.92
2851	94.98
2850	95.05
2849	95.12
2848	95.19
2847	95.25

2846	95.3
2845	95.35
2844	95.4
2843	95.46
2842	95.51
2841	95.56
2840	95.6
2839	95.64
2838	95.68
2837	95.73
2836	95.76
2835	95.8
2834	95.83
2833	95.85
2832	95.87
2831	95.89
2830	95.92
2829	95.95
2828	95.98
2827	96.01
2826	96.04
2825	96.06
2824	96.09
2823	96.12
2822	96.14
2821	96.16
2820	96.17
2819	96.19
2818	96.21
2817	96.24
2816	96.27
2815	96.3
2814	96.32
2813	96.33

2812	96.35
2811	96.36
2810	96.37
2809	96.39
2808	96.41
2807	96.43
2806	96.45
2805	96.47
2804	96.49
2803	96.51
2802	96.53
2801	96.55
2800	96.56
2799	96.57
2798	96.58
2797	96.59
2796	96.61
2795	96.63
2794	96.65
2793	96.66
2792	96.67
2791	96.69
2790	96.7
2789	96.71
2788	96.72
2787	96.73
2786	96.73
2785	96.74
2784	96.74
2783	96.75
2782	96.77
2781	96.78
2780	96.8
2779	96.82

2778	96.84
2777	96.85
2776	96.86
2775	96.87
2774	96.89
2773	96.91
2772	96.93
2771	96.95
2770	96.96
2769	96.97
2768	96.97
2767	96.97
2766	96.98
2765	96.99
2764	97
2763	97.01
2762	97.02
2761	97.02
2760	97.03
2759	97.05
2758	97.06
2757	97.08
2756	97.09
2755	97.1
2754	97.11
2753	97.11
2752	97.12
2751	97.12
2750	97.12
2749	97.13
2748	97.14
2747	97.15
2746	97.15
2745	97.15

2744	97.15
2743	97.16
2742	97.16
2741	97.17
2740	97.18
2739	97.19
2738	97.19
2737	97.2
2736	97.2
2735	97.2
2734	97.2
2733	97.2
2732	97.21
2731	97.21
2730	97.23
2729	97.24
2728	97.26
2727	97.27
2726	97.27
2725	97.27
2724	97.27
2723	97.27
2722	97.28
2721	97.3
2720	97.32
2719	97.33
2718	97.34
2717	97.35
2716	97.35
2715	97.36
2714	97.37
2713	97.38
2712	97.38
2711	97.38

2710	97.38
2709	97.38
2708	97.39
2707	97.39
2706	97.4
2705	97.41
2704	97.42
2703	97.43
2702	97.43
2701	97.44
2700	97.45
2699	97.46
2698	97.47
2697	97.47
2696	97.46
2695	97.45
2694	97.45
2693	97.45
2692	97.46
2691	97.47
2690	97.48
2689	97.49
2688	97.5
2687	97.51
2686	97.53
2685	97.55
2684	97.57
2683	97.58
2682	97.59
2681	97.59
2680	97.6
2679	97.61
2678	97.61
2677	97.62

2676	97.62
2675	97.62
2674	97.62
2673	97.63
2672	97.63
2671	97.63
2670	97.63
2669	97.63
2668	97.64
2667	97.65
2666	97.65
2665	97.64
2664	97.63
2663	97.62
2662	97.61
2661	97.61
2660	97.62
2659	97.64
2658	97.64
2657	97.65
2656	97.65
2655	97.64
2654	97.64
2653	97.64
2652	97.65
2651	97.66
2650	97.66
2649	97.67
2648	97.67
2647	97.67
2646	97.68
2645	97.69
2644	97.71
2643	97.72

2642	97.74
2641	97.75
2640	97.76
2639	97.76
2638	97.76
2637	97.76
2636	97.77
2635	97.78
2634	97.78
2633	97.79
2632	97.79
2631	97.8
2630	97.82
2629	97.83
2628	97.85
2627	97.87
2626	97.88
2625	97.89
2624	97.89
2623	97.89
2622	97.89
2621	97.89
2620	97.9
2619	97.9
2618	97.9
2617	97.9
2616	97.91
2615	97.91
2614	97.92
2613	97.93
2612	97.94
2611	97.95
2610	97.96
2609	97.97

2608	97.97
2607	97.96
2606	97.95
2605	97.95
2604	97.96
2603	97.98
2602	98
2601	98.03
2600	98.05
2599	98.07
2598	98.07
2597	98.07
2596	98.06
2595	98.06
2594	98.05
2593	98.05
2592	98.06
2591	98.07
2590	98.09
2589	98.1
2588	98.12
2587	98.12
2586	98.13
2585	98.13
2584	98.13
2583	98.14
2582	98.14
2581	98.13
2580	98.14
2579	98.14
2578	98.15
2577	98.16
2576	98.16
2575	98.16

2574	98.16
2573	98.15
2572	98.15
2571	98.17
2570	98.2
2569	98.23
2568	98.25
2567	98.25
2566	98.24
2565	98.23
2564	98.21
2563	98.2
2562	98.2
2561	98.2
2560	98.2
2559	98.2
2558	98.21
2557	98.23
2556	98.25
2555	98.27
2554	98.28
2553	98.3
2552	98.31
2551	98.32
2550	98.33
2549	98.34
2548	98.35
2547	98.36
2546	98.37
2545	98.37
2544	98.36
2543	98.35
2542	98.34
2541	98.33

2540	98.32
2539	98.32
2538	98.32
2537	98.32
2536	98.33
2535	98.33
2534	98.34
2533	98.35
2532	98.37
2531	98.39
2530	98.4
2529	98.39
2528	98.38
2527	98.37
2526	98.37
2525	98.38
2524	98.4
2523	98.41
2522	98.41
2521	98.4
2520	98.39
2519	98.4
2518	98.42
2517	98.43
2516	98.44
2515	98.43
2514	98.42
2513	98.42
2512	98.42
2511	98.43
2510	98.44
2509	98.44
2508	98.44
2507	98.44

2506	98.44
2505	98.45
2504	98.46
2503	98.48
2502	98.49
2501	98.5
2500	98.51
2499	98.51
2498	98.51
2497	98.51
2496	98.51
2495	98.53
2494	98.54
2493	98.56
2492	98.55
2491	98.54
2490	98.53
2489	98.54
2488	98.54
2487	98.54
2486	98.53
2485	98.54
2484	98.54
2483	98.56
2482	98.57
2481	98.58
2480	98.59
2479	98.6
2478	98.6
2477	98.6
2476	98.6
2475	98.59
2474	98.58
2473	98.57

2472	98.57
2471	98.59
2470	98.61
2469	98.63
2468	98.63
2467	98.62
2466	98.62
2465	98.63
2464	98.63
2463	98.63
2462	98.63
2461	98.63
2460	98.63
2459	98.63
2458	98.65
2457	98.67
2456	98.67
2455	98.66
2454	98.65
2453	98.63
2452	98.62
2451	98.63
2450	98.64
2449	98.66
2448	98.65
2447	98.64
2446	98.63
2445	98.63
2444	98.63
2443	98.62
2442	98.61
2441	98.61
2440	98.63
2439	98.66

2438	98.69
2437	98.71
2436	98.72
2435	98.72
2434	98.73
2433	98.73
2432	98.73
2431	98.73
2430	98.72
2429	98.72
2428	98.71
2427	98.72
2426	98.73
2425	98.74
2424	98.75
2423	98.75
2422	98.76
2421	98.77
2420	98.78
2419	98.78
2418	98.77
2417	98.77
2416	98.78
2415	98.79
2414	98.79
2413	98.79
2412	98.77
2411	98.76
2410	98.75
2409	98.75
2408	98.77
2407	98.79
2406	98.8
2405	98.79

2404	98.78
2403	98.78
2402	98.79
2401	98.81
2400	98.82
2399	98.81
2398	98.81
2397	98.82
2396	98.84
2395	98.86
2394	98.87
2393	98.86
2392	98.85
2391	98.83
2390	98.83
2389	98.83
2388	98.83
2387	98.83
2386	98.83
2385	98.83
2384	98.85
2383	98.86
2382	98.86
2381	98.86
2380	98.86
2379	98.86
2378	98.86
2377	98.86
2376	98.87
2375	98.87
2374	98.88
2373	98.89
2372	98.91
2371	98.93

2370	98.94
2369	98.94
2368	98.95
2367	98.96
2366	98.95
2365	98.91
2364	98.86
2363	98.81
2362	98.79
2361	98.79
2360	98.8
2359	98.82
2358	98.83
2357	98.83
2356	98.83
2355	98.84
2354	98.85
2353	98.85
2352	98.85
2351	98.84
2350	98.83
2349	98.82
2348	98.8
2347	98.78
2346	98.78
2345	98.79
2344	98.8
2343	98.79
2342	98.77
2341	98.74
2340	98.73
2339	98.73
2338	98.73
2337	98.75

2336	98.77
2335	98.78
2334	98.79
2333	98.8
2332	98.81
2331	98.82
2330	98.82
2329	98.79
2328	98.74
2327	98.66
2326	98.56
2325	98.49
2324	98.47
2323	98.51
2322	98.57
2321	98.62
2320	98.64
2319	98.66
2318	98.67
2317	98.69
2316	98.71
2315	98.72
2314	98.74
2313	98.75
2312	98.77
2311	98.78
2310	98.78
2309	98.76
2308	98.73
2307	98.7
2306	98.67
2305	98.65
2304	98.65
2303	98.66

2302	98.68
2301	98.7
2300	98.71
2299	98.7
2298	98.68
2297	98.67
2296	98.67
2295	98.67
2294	98.66
2293	98.64
2292	98.62
2291	98.61
2290	98.6
2289	98.6
2288	98.58
2287	98.57
2286	98.56
2285	98.57
2284	98.6
2283	98.63
2282	98.65
2281	98.66
2280	98.65
2279	98.64
2278	98.65
2277	98.66
2276	98.68
2275	98.68
2274	98.66
2273	98.65
2272	98.65
2271	98.68
2270	98.72
2269	98.76

2268	98.77
2267	98.75
2266	98.74
2265	98.73
2264	98.75
2263	98.78
2262	98.8
2261	98.82
2260	98.82
2259	98.81
2258	98.79
2257	98.78
2256	98.78
2255	98.78
2254	98.77
2253	98.77
2252	98.78
2251	98.8
2250	98.83
2249	98.85
2248	98.85
2247	98.83
2246	98.81
2245	98.79
2244	98.79
2243	98.78
2242	98.74
2241	98.69
2240	98.67
2239	98.7
2238	98.78
2237	98.86
2236	98.91
2235	98.91

2234	98.89
2233	98.86
2232	98.84
2231	98.81
2230	98.79
2229	98.79
2228	98.83
2227	98.89
2226	98.93
2225	98.93
2224	98.91
2223	98.9
2222	98.89
2221	98.87
2220	98.85
2219	98.85
2218	98.87
2217	98.88
2216	98.89
2215	98.89
2214	98.88
2213	98.88
2212	98.89
2211	98.92
2210	98.93
2209	98.91
2208	98.87
2207	98.85
2206	98.87
2205	98.91
2204	98.92
2203	98.91
2202	98.88
2201	98.85

2200	98.83
2199	98.82
2198	98.83
2197	98.86
2196	98.91
2195	98.95
2194	98.95
2193	98.88
2192	98.79
2191	98.73
2190	98.73
2189	98.78
2188	98.85
2187	98.93
2186	98.98
2185	99.01
2184	98.99
2183	98.94
2182	98.92
2181	98.95
2180	99.03
2179	99.11
2178	99.15
2177	99.14
2176	99.1
2175	99.06
2174	99.06
2173	99.11
2172	99.18
2171	99.24
2170	99.25
2169	99.21
2168	99.14
2167	99.07

2166	99
2165	98.98
2164	99
2163	99.07
2162	99.19
2161	99.36
2160	99.6
2159	99.9
2158	100.13
2157	100.2
2156	100.1
2155	99.92
2154	99.76
2153	99.67
2152	99.64
2151	99.63
2150	99.6
2149	99.54
2148	99.47
2147	99.4
2146	99.35
2145	99.34
2144	99.35
2143	99.35
2142	99.34
2141	99.35
2140	99.38
2139	99.4
2138	99.39
2137	99.39
2136	99.45
2135	99.55
2134	99.64
2133	99.64

2132	99.57
2131	99.48
2130	99.4
2129	99.35
2128	99.32
2127	99.29
2126	99.25
2125	99.21
2124	99.18
2123	99.16
2122	99.16
2121	99.16
2120	99.17
2119	99.17
2118	99.17
2117	99.14
2116	99.11
2115	99.07
2114	99.04
2113	99.03
2112	99.01
2111	99
2110	98.99
2109	98.98
2108	98.96
2107	98.96
2106	98.98
2105	99.02
2104	99.05
2103	99.07
2102	99.06
2101	99.04
2100	99.02
2099	99

2098	99.01
2097	99.07
2096	99.15
2095	99.23
2094	99.26
2093	99.24
2092	99.2
2091	99.15
2090	99.09
2089	99.04
2088	99.01
2087	99.01
2086	99.03
2085	99.04
2084	99.04
2083	99.05
2082	99.05
2081	99.07
2080	99.09
2079	99.12
2078	99.13
2077	99.12
2076	99.08
2075	99.04
2074	99.02
2073	99.03
2072	99.08
2071	99.13
2070	99.13
2069	99.1
2068	99.07
2067	99.06
2066	99.07
2065	99.09

2064	99.09
2063	99.12
2062	99.17
2061	99.25
2060	99.3
2059	99.3
2058	99.24
2057	99.17
2056	99.15
2055	99.18
2054	99.21
2053	99.18
2052	99.09
2051	99.01
2050	99
2049	99.06
2048	99.13
2047	99.16
2046	99.16
2045	99.16
2044	99.21
2043	99.29
2042	99.37
2041	99.4
2040	99.4
2039	99.39
2038	99.4
2037	99.44
2036	99.54
2035	99.66
2034	99.74
2033	99.72
2032	99.64
2031	99.56

2030	99.52
2029	99.52
2028	99.55
2027	99.61
2026	99.69
2025	99.77
2024	99.83
2023	99.84
2022	99.82
2021	99.78
2020	99.74
2019	99.7
2018	99.68
2017	99.68
2016	99.72
2015	99.8
2014	99.87
2013	99.92
2012	99.96
2011	100
2010	100
2009	99.95
2008	99.89
2007	99.87
2006	99.86
2005	99.84
2004	99.78
2003	99.71
2002	99.65
2001	99.61
2000	99.57
1999	99.54
1998	99.49
1997	99.44

1996	99.4
1995	99.38
1994	99.38
1993	99.38
1992	99.36
1991	99.32
1990	99.27
1989	99.22
1988	99.2
1987	99.21
1986	99.27
1985	99.33
1984	99.37
1983	99.38
1982	99.36
1981	99.34
1980	99.39
1979	99.52
1978	99.7
1977	99.85
1976	99.92
1975	99.94
1974	99.93
1973	99.92
1972	99.91
1971	99.9
1970	99.89
1969	99.89
1968	99.9
1967	99.92
1966	99.89
1965	99.8
1964	99.67
1963	99.58

1962	99.59
1961	99.7
1960	99.85
1959	99.97
1958	100.01
1957	99.95
1956	99.85
1955	99.74
1954	99.63
1953	99.55
1952	99.51
1951	99.52
1950	99.56
1949	99.59
1948	99.61
1947	99.59
1946	99.55
1945	99.5
1944	99.49
1943	99.52
1942	99.53
1941	99.5
1940	99.45
1939	99.42
1938	99.43
1937	99.45
1936	99.47
1935	99.48
1934	99.49
1933	99.49
1932	99.49
1931	99.5
1930	99.52
1929	99.52

1928	99.49
1927	99.45
1926	99.4
1925	99.37
1924	99.34
1923	99.35
1922	99.37
1921	99.38
1920	99.38
1919	99.38
1918	99.4
1917	99.42
1916	99.43
1915	99.42
1914	99.41
1913	99.42
1912	99.43
1911	99.41
1910	99.38
1909	99.34
1908	99.33
1907	99.34
1906	99.38
1905	99.39
1904	99.38
1903	99.37
1902	99.39
1901	99.43
1900	99.46
1899	99.45
1898	99.42
1897	99.37
1896	99.35
1895	99.33

1894	99.32
1893	99.31
1892	99.31
1891	99.32
1890	99.34
1889	99.37
1888	99.39
1887	99.4
1886	99.38
1885	99.37
1884	99.37
1883	99.39
1882	99.4
1881	99.37
1880	99.32
1879	99.27
1878	99.25
1877	99.25
1876	99.25
1875	99.25
1874	99.24
1873	99.24
1872	99.25
1871	99.28
1870	99.3
1869	99.31
1868	99.3
1867	99.27
1866	99.26
1865	99.26
1864	99.28
1863	99.3
1862	99.3
1861	99.29

1860	99.29
1859	99.3
1858	99.32
1857	99.34
1856	99.34
1855	99.34
1854	99.33
1853	99.31
1852	99.29
1851	99.27
1850	99.26
1849	99.25
1848	99.25
1847	99.24
1846	99.22
1845	99.2
1844	99.19
1843	99.22
1842	99.26
1841	99.3
1840	99.33
1839	99.34
1838	99.32
1837	99.29
1836	99.26
1835	99.25
1834	99.25
1833	99.25
1832	99.26
1831	99.28
1830	99.29
1829	99.29
1828	99.28
1827	99.27

1826	99.26
1825	99.26
1824	99.25
1823	99.23
1822	99.21
1821	99.2
1820	99.2
1819	99.22
1818	99.25
1817	99.26
1816	99.26
1815	99.25
1814	99.24
1813	99.23
1812	99.23
1811	99.21
1810	99.19
1809	99.17
1808	99.16
1807	99.16
1806	99.17
1805	99.17
1804	99.17
1803	99.17
1802	99.16
1801	99.15
1800	99.13
1799	99.11
1798	99.1
1797	99.1
1796	99.11
1795	99.11
1794	99.11
1793	99.12

1792	99.13
1791	99.14
1790	99.14
1789	99.13
1788	99.12
1787	99.12
1786	99.11
1785	99.1
1784	99.08
1783	99.06
1782	99.05
1781	99.04
1780	99.04
1779	99.04
1778	99.05
1777	99.08
1776	99.1
1775	99.11
1774	99.09
1773	99.07
1772	99.05
1771	99.04
1770	99.04
1769	99.04
1768	99.04
1767	99.04
1766	99.04
1765	99.02
1764	99.01
1763	99
1762	99
1761	99
1760	98.99
1759	98.98

1758	98.98
1757	98.98
1756	98.99
1755	98.98
1754	98.97
1753	98.94
1752	98.91
1751	98.89
1750	98.88
1749	98.88
1748	98.87
1747	98.85
1746	98.82
1745	98.81
1744	98.81
1743	98.81
1742	98.8
1741	98.77
1740	98.74
1739	98.72
1738	98.7
1737	98.68
1736	98.65
1735	98.62
1734	98.62
1733	98.64
1732	98.65
1731	98.66
1730	98.67
1729	98.66
1728	98.63
1727	98.59
1726	98.56
1725	98.52

1724	98.48
1723	98.43
1722	98.38
1721	98.34
1720	98.3
1719	98.29
1718	98.31
1717	98.34
1716	98.36
1715	98.37
1714	98.38
1713	98.38
1712	98.38
1711	98.37
1710	98.35
1709	98.32
1708	98.29
1707	98.27
1706	98.27
1705	98.26
1704	98.26
1703	98.24
1702	98.21
1701	98.15
1700	98.09
1699	98.07
1698	98.06
1697	98.05
1696	98.02
1695	97.96
1694	97.91
1693	97.85
1692	97.8
1691	97.75

1690	97.68
1689	97.62
1688	97.56
1687	97.53
1686	97.48
1685	97.36
1684	97.19
1683	97.06
1682	96.98
1681	96.91
1680	96.83
1679	96.74
1678	96.65
1677	96.56
1676	96.43
1675	96.29
1674	96.18
1673	96.09
1672	95.99
1671	95.85
1670	95.69
1669	95.56
1668	95.47
1667	95.43
1666	95.38
1665	95.32
1664	95.22
1663	95.1
1662	94.99
1661	94.91
1660	94.85
1659	94.8
1658	94.76
1657	94.74

1656	94.73
1655	94.73
1654	94.68
1653	94.59
1652	94.53
1651	94.52
1650	94.52
1649	94.51
1648	94.49
1647	94.46
1646	94.46
1645	94.49
1644	94.51
1643	94.53
1642	94.54
1641	94.55
1640	94.57
1639	94.59
1638	94.61
1637	94.62
1636	94.64
1635	94.67
1634	94.72
1633	94.76
1632	94.8
1631	94.83
1630	94.86
1629	94.9
1628	94.94
1627	94.98
1626	95.03
1625	95.06
1624	95.08
1623	95.1

1622	95.13
1621	95.16
1620	95.18
1619	95.2
1618	95.21
1617	95.2
1616	95.18
1615	95.15
1614	95.13
1613	95.11
1612	95.08
1611	95.05
1610	95.01
1609	94.97
1608	94.93
1607	94.88
1606	94.83
1605	94.78
1604	94.71
1603	94.64
1602	94.57
1601	94.52
1600	94.47
1599	94.43
1598	94.4
1597	94.35
1596	94.3
1595	94.24
1594	94.19
1593	94.14
1592	94.09
1591	94.04
1590	94.01
1589	93.99

1588	93.99
1587	93.98
1586	93.97
1585	93.96
1584	93.96
1583	93.97
1582	93.98
1581	93.99
1580	94
1579	94.02
1578	94.05
1577	94.07
1576	94.09
1575	94.12
1574	94.17
1573	94.22
1572	94.27
1571	94.3
1570	94.34
1569	94.38
1568	94.44
1567	94.5
1566	94.56
1565	94.6
1564	94.63
1563	94.66
1562	94.72
1561	94.79
1560	94.82
1559	94.79
1558	94.78
1557	94.82
1556	94.87
1555	94.92

1554	94.97
1553	95.02
1552	95.07
1551	95.12
1550	95.16
1549	95.18
1548	95.2
1547	95.22
1546	95.24
1545	95.25
1544	95.24
1543	95.22
1542	95.22
1541	95.28
1540	95.4
1539	95.53
1538	95.6
1537	95.64
1536	95.67
1535	95.69
1534	95.73
1533	95.79
1532	95.85
1531	95.91
1530	95.96
1529	96.02
1528	96.09
1527	96.15
1526	96.19
1525	96.22
1524	96.23
1523	96.26
1522	96.3
1521	96.37

1520	96.44
1519	96.49
1518	96.53
1517	96.56
1516	96.59
1515	96.61
1514	96.64
1513	96.66
1512	96.68
1511	96.7
1510	96.71
1509	96.72
1508	96.71
1507	96.75
1506	96.81
1505	96.82
1504	96.81
1503	96.8
1502	96.79
1501	96.79
1500	96.78
1499	96.79
1498	96.81
1497	96.82
1496	96.82
1495	96.8
1494	96.78
1493	96.77
1492	96.75
1491	96.74
1490	96.72
1489	96.7
1488	96.67
1487	96.64

1486	96.61
1485	96.59
1484	96.56
1483	96.53
1482	96.48
1481	96.43
1480	96.37
1479	96.33
1478	96.29
1477	96.23
1476	96.17
1475	96.11
1474	96.05
1473	95.99
1472	95.92
1471	95.86
1470	95.82
1469	95.77
1468	95.73
1467	95.68
1466	95.61
1465	95.54
1464	95.48
1463	95.46
1462	95.45
1461	95.44
1460	95.43
1459	95.41
1458	95.36
1457	95.32
1456	95.3
1455	95.29
1454	95.28
1453	95.27

1452	95.26
1451	95.25
1450	95.24
1449	95.23
1448	95.21
1447	95.19
1446	95.18
1445	95.16
1444	95.14
1443	95.11
1442	95.08
1441	95.04
1440	95
1439	94.96
1438	94.91
1437	94.83
1436	94.75
1435	94.69
1434	94.65
1433	94.62
1432	94.57
1431	94.52
1430	94.45
1429	94.38
1428	94.32
1427	94.28
1426	94.24
1425	94.19
1424	94.14
1423	94.1
1422	94.09
1421	94.1
1420	94.12
1419	94.13

1418	94.15
1417	94.16
1416	94.2
1415	94.25
1414	94.32
1413	94.39
1412	94.46
1411	94.53
1410	94.6
1409	94.66
1408	94.73
1407	94.8
1406	94.86
1405	94.9
1404	94.93
1403	94.95
1402	94.97
1401	94.97
1400	94.95
1399	94.9
1398	94.84
1397	94.76
1396	94.67
1395	94.55
1394	94.44
1393	94.34
1392	94.24
1391	94.15
1390	94.05
1389	93.94
1388	93.81
1387	93.67
1386	93.54
1385	93.42

1384	93.3
1383	93.17
1382	93.04
1381	92.93
1380	92.83
1379	92.77
1378	92.73
1377	92.71
1376	92.69
1375	92.7
1374	92.73
1373	92.78
1372	92.84
1371	92.93
1370	93.04
1369	93.18
1368	93.3
1367	93.41
1366	93.51
1365	93.59
1364	93.67
1363	93.73
1362	93.79
1361	93.84
1360	93.89
1359	93.94
1358	93.98
1357	94.02
1356	94.04
1355	94.05
1354	94.05
1353	94.03
1352	94.01
1351	93.99

1350	93.97
1349	93.96
1348	93.96
1347	93.97
1346	93.98
1345	94
1344	94.01
1343	94.04
1342	94.07
1341	94.1
1340	94.11
1339	94.11
1338	94.1
1337	94.1
1336	94.1
1335	94.11
1334	94.13
1333	94.14
1332	94.14
1331	94.12
1330	94.08
1329	94.03
1328	93.97
1327	93.92
1326	93.88
1325	93.85
1324	93.82
1323	93.8
1322	93.79
1321	93.78
1320	93.79
1319	93.82
1318	93.86
1317	93.92

1316	93.97
1315	94.01
1314	94.06
1313	94.09
1312	94.12
1311	94.15
1310	94.18
1309	94.2
1308	94.23
1307	94.26
1306	94.3
1305	94.34
1304	94.37
1303	94.4
1302	94.44
1301	94.46
1300	94.49
1299	94.51
1298	94.54
1297	94.57
1296	94.6
1295	94.63
1294	94.66
1293	94.7
1292	94.75
1291	94.79
1290	94.82
1289	94.85
1288	94.89
1287	94.93
1286	94.97
1285	95.02
1284	95.06
1283	95.12

1282	95.2
1281	95.29
1280	95.38
1279	95.44
1278	95.49
1277	95.52
1276	95.53
1275	95.54
1274	95.53
1273	95.51
1272	95.49
1271	95.47
1270	95.46
1269	95.46
1268	95.46
1267	95.46
1266	95.45
1265	95.44
1264	95.42
1263	95.41
1262	95.38
1261	95.36
1260	95.34
1259	95.33
1258	95.33
1257	95.31
1256	95.3
1255	95.28
1254	95.26
1253	95.24
1252	95.24
1251	95.25
1250	95.27
1249	95.29

1248	95.32
1247	95.36
1246	95.42
1245	95.49
1244	95.57
1243	95.65
1242	95.72
1241	95.78
1240	95.84
1239	95.88
1238	95.9
1237	95.93
1236	95.95
1235	95.98
1234	96
1233	96.02
1232	96.04
1231	96.06
1230	96.08
1229	96.11
1228	96.15
1227	96.19
1226	96.23
1225	96.26
1224	96.3
1223	96.34
1222	96.38
1221	96.42
1220	96.45
1219	96.5
1218	96.54
1217	96.57
1216	96.6
1215	96.61

1214	96.62
1213	96.63
1212	96.65
1211	96.65
1210	96.64
1209	96.61
1208	96.57
1207	96.52
1206	96.47
1205	96.41
1204	96.34
1203	96.26
1202	96.19
1201	96.13
1200	96.1
1199	96.08
1198	96.08
1197	96.07
1196	96.07
1195	96.07
1194	96.07
1193	96.08
1192	96.11
1191	96.16
1190	96.23
1189	96.31
1188	96.39
1187	96.47
1186	96.53
1185	96.57
1184	96.58
1183	96.56
1182	96.52
1181	96.46

1180	96.4
1179	96.33
1178	96.27
1177	96.2
1176	96.11
1175	96
1174	95.86
1173	95.7
1172	95.54
1171	95.4
1170	95.26
1169	95.11
1168	94.96
1167	94.78
1166	94.58
1165	94.35
1164	94.12
1163	93.86
1162	93.57
1161	93.26
1160	92.93
1159	92.6
1158	92.29
1157	91.99
1156	91.7
1155	91.43
1154	91.19
1153	90.99
1152	90.81
1151	90.68
1150	90.61
1149	90.62
1148	90.68
1147	90.81

1146	90.99
1145	91.21
1144	91.46
1143	91.73
1142	91.98
1141	92.22
1140	92.41
1139	92.56
1138	92.67
1137	92.74
1136	92.8
1135	92.84
1134	92.85
1133	92.81
1132	92.72
1131	92.6
1130	92.46
1129	92.31
1128	92.16
1127	92
1126	91.86
1125	91.73
1124	91.61
1123	91.48
1122	91.34
1121	91.18
1120	90.99
1119	90.81
1118	90.64
1117	90.5
1116	90.39
1115	90.32
1114	90.26
1113	90.18

1112	90.07
1111	89.93
1110	89.76
1109	89.56
1108	89.32
1107	89.06
1106	88.77
1105	88.45
1104	88.11
1103	87.74
1102	87.34
1101	86.91
1100	86.49
1099	86.1
1098	85.75
1097	85.45
1096	85.16
1095	84.9
1094	84.68
1093	84.47
1092	84.27
1091	84.07
1090	83.87
1089	83.65
1088	83.41
1087	83.17
1086	82.93
1085	82.7
1084	82.46
1083	82.25
1082	82.06
1081	81.92
1080	81.82
1079	81.77

1078	81.75
1077	81.74
1076	81.76
1075	81.81
1074	81.88
1073	81.95
1072	82.01
1071	82.06
1070	82.09
1069	82.1
1068	82.08
1067	82.07
1066	82.07
1065	82.06
1064	82.04
1063	82
1062	81.94
1061	81.88
1060	81.83
1059	81.8
1058	81.77
1057	81.74
1056	81.71
1055	81.7
1054	81.72
1053	81.76
1052	81.8
1051	81.86
1050	81.93
1049	81.96
1048	81.95
1047	81.9
1046	81.83
1045	81.73

1044	81.61
1043	81.48
1042	81.31
1041	81.12
1040	80.91
1039	80.69
1038	80.47
1037	80.25
1036	80.01
1035	79.79
1034	79.6
1033	79.45
1032	79.36
1031	79.33
1030	79.35
1029	79.4
1028	79.46
1027	79.53
1026	79.6
1025	79.66
1024	79.72
1023	79.79
1022	79.88
1021	79.97
1020	80.07
1019	80.19
1018	80.32
1017	80.45
1016	80.57
1015	80.71
1014	80.85
1013	80.98
1012	81.09
1011	81.19

1010	81.28
1009	81.35
1008	81.42
1007	81.49
1006	81.57
1005	81.63
1004	81.68
1003	81.71
1002	81.75
1001	81.78
1000	81.81
999	81.83
998	81.81
997	81.75
996	81.66
995	81.58
994	81.52
993	81.48
992	81.48
991	81.52
990	81.58
989	81.65
988	81.7
987	81.74
986	81.81
985	81.9
984	82.01
983	82.15
982	82.3
981	82.45
980	82.59
979	82.74
978	82.91
977	83.09

976	83.28
975	83.48
974	83.68
973	83.88
972	84.05
971	84.19
970	84.32
969	84.46
968	84.6
967	84.74
966	84.87
965	84.98
964	85.09
963	85.18
962	85.25
961	85.29
960	85.3
959	85.31
958	85.34
957	85.38
956	85.45
955	85.53
954	85.59
953	85.62
952	85.62
951	85.62
950	85.62
949	85.62
948	85.6
947	85.58
946	85.57
945	85.57
944	85.57
943	85.58

942	85.59
941	85.62
940	85.66
939	85.71
938	85.78
937	85.84
936	85.9
935	85.97
934	86.07
933	86.18
932	86.3
931	86.41
930	86.52
929	86.63
928	86.75
927	86.88
926	87.02
925	87.16
924	87.31
923	87.48
922	87.64
921	87.79
920	87.93
919	88.06
918	88.18
917	88.27
916	88.35
915	88.41
914	88.46
913	88.5
912	88.56
911	88.61
910	88.63
909	88.6

908	88.51
907	88.41
906	88.31
905	88.21
904	88.11
903	88
902	87.88
901	87.74
900	87.58
899	87.43
898	87.32
897	87.25
896	87.21
895	87.2
894	87.19
893	87.19
892	87.21
891	87.25
890	87.34
889	87.45
888	87.59
887	87.73
886	87.88
885	88.04
884	88.2
883	88.36
882	88.53
881	88.69
880	88.85
879	89.02
878	89.18
877	89.32
876	89.45
875	89.55

874	89.64
873	89.72
872	89.8
871	89.89
870	90.01
869	90.12
868	90.22
867	90.3
866	90.36
865	90.39
864	90.42
863	90.44
862	90.47
861	90.5
860	90.55
859	90.6
858	90.64
857	90.67
856	90.7
855	90.73
854	90.74
853	90.73
852	90.71
851	90.71
850	90.75
849	90.82
848	90.88
847	90.91
846	90.91
845	90.9
844	90.89
843	90.89
842	90.91
841	90.92

840	90.91
839	90.87
838	90.81
837	90.74
836	90.7
835	90.7
834	90.74
833	90.8
832	90.84
831	90.85
830	90.83
829	90.79
828	90.76
827	90.77
826	90.77
825	90.77
824	90.75
823	90.72
822	90.7
821	90.67
820	90.62
819	90.58
818	90.54
817	90.51
816	90.49
815	90.49
814	90.5
813	90.5
812	90.49
811	90.45
810	90.39
809	90.31
808	90.24
807	90.19

806	90.16
805	90.14
804	90.13
803	90.12
802	90.12
801	90.11
800	90.08
799	90.03
798	89.99
797	89.95
796	89.91
795	89.85
794	89.79
793	89.74
792	89.72
791	89.72
790	89.73
789	89.73
788	89.73
787	89.71
786	89.69
785	89.65
784	89.61
783	89.58
782	89.54
781	89.5
780	89.46
779	89.41
778	89.37
777	89.34
776	89.33
775	89.35
774	89.38
773	89.37

772	89.33
771	89.28
770	89.23
769	89.19
768	89.16
767	89.15
766	89.14
765	89.11
764	89.05
763	88.98
762	88.92
761	88.87
760	88.83
759	88.82
758	88.82
757	88.8
756	88.78
755	88.77
754	88.77
753	88.76
752	88.74
751	88.73
750	88.74
749	88.76
748	88.77
747	88.76
746	88.72
745	88.65
744	88.59
743	88.56
742	88.55
741	88.53
740	88.5
739	88.46

738	88.42
737	88.37
736	88.32
735	88.27
734	88.26
733	88.27
732	88.29
731	88.32
730	88.35
729	88.37
728	88.35
727	88.31
726	88.27
725	88.25
724	88.23
723	88.21
722	88.17
721	88.12
720	88.06
719	87.99
718	87.95
717	87.93
716	87.91
715	87.86
714	87.79
713	87.71
712	87.67
711	87.67
710	87.73
709	87.78
708	87.79
707	87.75
706	87.69
705	87.64

704	87.63
703	87.65
702	87.68
701	87.68
700	87.64
699	87.59
698	87.55
697	87.53
696	87.53
695	87.54
694	87.54
693	87.54
692	87.55
691	87.57
690	87.61
689	87.63
688	87.63
687	87.6
686	87.57
685	87.52
684	87.47
683	87.42
682	87.36
681	87.27
680	87.15
679	87.01
678	86.87
677	86.72
676	86.55
675	86.37
674	86.19
673	86.04
672	85.95
671	85.9

670	85.83
669	85.62
668	85.24
667	84.89
666	84.7
665	84.59
664	84.49
663	84.39
662	84.3
661	84.26
660	84.26
659	84.31
658	84.4
657	84.5
656	84.59
655	84.66
654	84.72
653	84.75
652	84.78
651	84.82
650	84.87
649	84.92
648	84.94
647	84.94
646	84.89
645	84.79
644	84.69
643	84.64
642	84.63
641	84.61
640	84.55
639	84.49
638	84.46
637	84.46

636	84.45
635	84.4
634	84.3
633	84.2
632	84.11
631	84.07
630	84.04
629	83.99
628	83.94
627	83.91
626	83.93
625	83.98
624	84.03
623	84.05
622	84.03
621	83.95
620	83.85
619	83.76
618	83.72
617	83.69
616	83.66
615	83.64
614	83.62
613	83.58
612	83.52
611	83.48
610	83.45
609	83.43
608	83.41
607	83.36
606	83.29
605	83.2
604	83.09
603	83.01
