# Supplementary material for: Biotechnology-driven extraction and characterisation of Chitosan from the West African river prawn (Macrobrachium vollenhovenii) and American Cockroach (Periplaneta americana) using a modified approach
Source: PLoS One. 2026 May 28;21(5):e0349133. doi: 10.1371/journal.pone.0349133 (PMC13218523; doi:10.1371/journal.pone.0349133)
Supplement: S1 File — (ZIP) [file pone.0349133.s001.zip › PrawnSpectraValues.pdf]

Created as	Sample 016	June 20 2023
cm-1	%T	
4000	99.76	
3999	99.74	
3998	99.77	
3997	99.77	
3996	99.77	
3995	99.77	
3994	99.76	
3993	99.75	
3992	99.74	
3991	99.73	
3990	99.72	
3989	99.72	
3988	99.73	
3987	99.73	
3986	99.73	
3985	99.74	
3984	99.74	
3983	99.74	
3982	99.75	
3981	99.75	
3980	99.75	
3979	99.75	
3978	99.75	
3977	99.74	
3976	99.74	
3975	99.74	
3974	99.74	
3973	99.75	
3972	99.76	
3971	99.76	
3970	99.76	
3969	99.76	

3968	99.76
3967	99.76
3966	99.75
3965	99.75
3964	99.74
3963	99.73
3962	99.74
3961	99.74
3960	99.75
3959	99.75
3958	99.75
3957	99.75
3956	99.74
3955	99.74
3954	99.73
3953	99.73
3952	99.71
3951	99.69
3950	99.66
3949	99.64
3948	99.64
3947	99.65
3946	99.67
3945	99.71
3944	99.75
3943	99.77
3942	99.78
3941	99.77
3940	99.77
3939	99.76
3938	99.75
3937	99.73
3936	99.7
3935	99.67

3934	99.65
3933	99.65
3932	99.67
3931	99.7
3930	99.73
3929	99.76
3928	99.78
3927	99.79
3926	99.79
3925	99.78
3924	99.76
3923	99.75
3922	99.73
3921	99.72
3920	99.7
3919	99.68
3918	99.69
3917	99.71
3916	99.75
3915	99.77
3914	99.79
3913	99.79
3912	99.79
3911	99.79
3910	99.78
3909	99.76
3908	99.74
3907	99.71
3906	99.67
3905	99.62
3904	99.58
3903	99.56
3902	99.59
3901	99.66

3900	99.72
3899	99.76
3898	99.76
3897	99.75
3896	99.73
3895	99.72
3894	99.71
3893	99.72
3892	99.74
3891	99.73
3890	99.71
3889	99.68
3888	99.67
3887	99.68
3886	99.69
3885	99.7
3884	99.72
3883	99.75
3882	99.8
3881	99.84
3880	99.83
3879	99.79
3878	99.76
3877	99.76
3876	99.78
3875	99.79
3874	99.77
3873	99.72
3872	99.68
3871	99.68
3870	99.68
3869	99.66
3868	99.64
3867	99.62

3866	99.64
3865	99.67
3864	99.7
3863	99.71
3862	99.7
3861	99.69
3860	99.68
3859	99.67
3858	99.67
3857	99.64
3856	99.6
3855	99.56
3854	99.56
3853	99.59
3852	99.64
3851	99.68
3850	99.7
3849	99.71
3848	99.71
3847	99.72
3846	99.73
3845	99.75
3844	99.76
3843	99.75
3842	99.74
3841	99.73
3840	99.74
3839	99.76
3838	99.79
3837	99.79
3836	99.77
3835	99.75
3834	99.73
3833	99.72

3832	99.72
3831	99.72
3830	99.73
3829	99.75
3828	99.77
3827	99.77
3826	99.74
3825	99.68
3824	99.63
3823	99.6
3822	99.62
3821	99.64
3820	99.67
3819	99.7
3818	99.75
3817	99.8
3816	99.83
3815	99.83
3814	99.81
3813	99.79
3812	99.76
3811	99.72
3810	99.68
3809	99.65
3808	99.64
3807	99.63
3806	99.63
3805	99.63
3804	99.66
3803	99.7
3802	99.74
3801	99.74
3800	99.72
3799	99.71

3798	99.71
3797	99.74
3796	99.76
3795	99.77
3794	99.78
3793	99.8
3792	99.8
3791	99.79
3790	99.77
3789	99.75
3788	99.73
3787	99.73
3786	99.73
3785	99.73
3784	99.73
3783	99.72
3782	99.73
3781	99.75
3780	99.78
3779	99.79
3778	99.78
3777	99.77
3776	99.74
3775	99.72
3774	99.69
3773	99.66
3772	99.64
3771	99.63
3770	99.63
3769	99.62
3768	99.62
3767	99.62
3766	99.62
3765	99.62

3764	99.62
3763	99.61
3762	99.62
3761	99.62
3760	99.62
3759	99.61
3758	99.59
3757	99.56
3756	99.53
3755	99.5
3754	99.46
3753	99.39
3752	99.29
3751	99.21
3750	99.18
3749	99.19
3748	99.22
3747	99.27
3746	99.33
3745	99.37
3744	99.37
3743	99.36
3742	99.35
3741	99.34
3740	99.33
3739	99.33
3738	99.33
3737	99.34
3736	99.37
3735	99.4
3734	99.4
3733	99.39
3732	99.36
3731	99.35

3730	99.33
3729	99.32
3728	99.3
3727	99.29
3726	99.28
3725	99.29
3724	99.32
3723	99.35
3722	99.38
3721	99.4
3720	99.41
3719	99.4
3718	99.39
3717	99.36
3716	99.32
3715	99.28
3714	99.24
3713	99.2
3712	99.18
3711	99.19
3710	99.21
3709	99.22
3708	99.21
3707	99.2
3706	99.18
3705	99.17
3704	99.16
3703	99.15
3702	99.14
3701	99.11
3700	99.06
3699	99.02
3698	98.98
3697	98.94

3696	98.9
3695	98.86
3694	98.84
3693	98.83
3692	98.83
3691	98.83
3690	98.82
3689	98.79
3688	98.74
3687	98.69
3686	98.65
3685	98.62
3684	98.58
3683	98.54
3682	98.5
3681	98.47
3680	98.43
3679	98.37
3678	98.31
3677	98.25
3676	98.18
3675	98.09
3674	98
3673	97.94
3672	97.89
3671	97.86
3670	97.82
3669	97.76
3668	97.7
3667	97.64
3666	97.59
3665	97.55
3664	97.5
3663	97.45

3662	97.39
3661	97.31
3660	97.22
3659	97.13
3658	97.03
3657	96.93
3656	96.83
3655	96.76
3654	96.71
3653	96.68
3652	96.62
3651	96.51
3650	96.36
3649	96.25
3648	96.19
3647	96.14
3646	96.08
3645	96.01
3644	95.94
3643	95.88
3642	95.82
3641	95.76
3640	95.7
3639	95.64
3638	95.57
3637	95.51
3636	95.44
3635	95.35
3634	95.24
3633	95.12
3632	94.99
3631	94.86
3630	94.72
3629	94.6

3628	94.52
3627	94.49
3626	94.48
3625	94.46
3624	94.43
3623	94.39
3622	94.35
3621	94.29
3620	94.21
3619	94.1
3618	93.98
3617	93.87
3616	93.78
3615	93.72
3614	93.65
3613	93.58
3612	93.49
3611	93.4
3610	93.32
3609	93.27
3608	93.24
3607	93.2
3606	93.15
3605	93.08
3604	93.01
3603	92.94
3602	92.86
3601	92.78
3600	92.7
3599	92.63
3598	92.58
3597	92.51
3596	92.43
3595	92.34

3594	92.24
3593	92.15
3592	92.07
3591	92
3590	91.92
3589	91.83
3588	91.73
3587	91.63
3586	91.55
3585	91.49
3584	91.45
3583	91.42
3582	91.38
3581	91.33
3580	91.27
3579	91.21
3578	91.15
3577	91.08
3576	91.02
3575	90.95
3574	90.88
3573	90.82
3572	90.76
3571	90.72
3570	90.68
3569	90.62
3568	90.55
3567	90.48
3566	90.42
3565	90.37
3564	90.33
3563	90.28
3562	90.23
3561	90.16

3560	90.08
3559	89.97
3558	89.85
3557	89.73
3556	89.6
3555	89.46
3554	89.31
3553	89.16
3552	89
3551	88.86
3550	88.72
3549	88.58
3548	88.43
3547	88.27
3546	88.13
3545	88.03
3544	87.95
3543	87.9
3542	87.84
3541	87.77
3540	87.68
3539	87.57
3538	87.44
3537	87.32
3536	87.22
3535	87.13
3534	87.05
3533	86.96
3532	86.86
3531	86.76
3530	86.65
3529	86.52
3528	86.4
3527	86.27

3526	86.15
3525	86.03
3524	85.94
3523	85.85
3522	85.77
3521	85.69
3520	85.59
3519	85.49
3518	85.37
3517	85.25
3516	85.13
3515	85.01
3514	84.9
3513	84.79
3512	84.66
3511	84.53
3510	84.39
3509	84.25
3508	84.11
3507	83.96
3506	83.8
3505	83.62
3504	83.43
3503	83.24
3502	83.08
3501	82.92
3500	82.76
3499	82.59
3498	82.39
3497	82.17
3496	81.96
3495	81.75
3494	81.55
3493	81.35

3492	81.15
3491	80.94
3490	80.73
3489	80.52
3488	80.31
3487	80.13
3486	79.96
3485	79.81
3484	79.65
3483	79.5
3482	79.34
3481	79.2
3480	79.09
3479	79
3478	78.93
3477	78.85
3476	78.78
3475	78.7
3474	78.61
3473	78.52
3472	78.42
3471	78.32
3470	78.21
3469	78.1
3468	78.01
3467	77.93
3466	77.85
3465	77.77
3464	77.66
3463	77.54
3462	77.41
3461	77.28
3460	77.15
3459	77.03

3458	76.91
3457	76.79
3456	76.67
3455	76.55
3454	76.42
3453	76.29
3452	76.16
3451	76.02
3450	75.89
3449	75.75
3448	75.62
3447	75.49
3446	75.37
3445	75.27
3444	75.19
3443	75.11
3442	75.03
3441	74.95
3440	74.86
3439	74.78
3438	74.71
3437	74.66
3436	74.63
3435	74.61
3434	74.61
3433	74.6
3432	74.59
3431	74.59
3430	74.6
3429	74.61
3428	74.64
3427	74.67
3426	74.69
3425	74.71

3424	74.73
3423	74.75
3422	74.76
3421	74.78
3420	74.8
3419	74.83
3418	74.85
3417	74.87
3416	74.88
3415	74.88
3414	74.88
3413	74.89
3412	74.9
3411	74.91
3410	74.93
3409	74.94
3408	74.95
3407	74.97
3406	74.97
3405	74.97
3404	74.95
3403	74.94
3402	74.92
3401	74.92
3400	74.92
3399	74.92
3398	74.92
3397	74.92
3396	74.9
3395	74.88
3394	74.86
3393	74.83
3392	74.82
3391	74.81

3390	74.83
3389	74.84
3388	74.85
3387	74.86
3386	74.85
3385	74.84
3384	74.83
3383	74.81
3382	74.8
3381	74.8
3380	74.79
3379	74.79
3378	74.8
3377	74.81
3376	74.82
3375	74.83
3374	74.84
3373	74.83
3372	74.82
3371	74.82
3370	74.82
3369	74.83
3368	74.84
3367	74.86
3366	74.89
3365	74.91
3364	74.93
3363	74.94
3362	74.94
3361	74.96
3360	74.99
3359	75.03
3358	75.07
3357	75.1

3356	75.12
3355	75.14
3354	75.15
3353	75.16
3352	75.17
3351	75.19
3350	75.21
3349	75.24
3348	75.27
3347	75.29
3346	75.31
3345	75.33
3344	75.34
3343	75.36
3342	75.38
3341	75.39
3340	75.39
3339	75.4
3338	75.4
3337	75.41
3336	75.43
3335	75.44
3334	75.46
3333	75.47
3332	75.47
3331	75.48
3330	75.48
3329	75.47
3328	75.47
3327	75.45
3326	75.43
3325	75.4
3324	75.37
3323	75.34

3322	75.31
3321	75.28
3320	75.25
3319	75.21
3318	75.16
3317	75.12
3316	75.07
3315	75.03
3314	74.99
3313	74.96
3312	74.92
3311	74.88
3310	74.83
3309	74.78
3308	74.74
3307	74.68
3306	74.63
3305	74.56
3304	74.5
3303	74.43
3302	74.36
3301	74.3
3300	74.22
3299	74.15
3298	74.08
3297	74.01
3296	73.94
3295	73.86
3294	73.78
3293	73.7
3292	73.61
3291	73.52
3290	73.43
3289	73.34

3288	73.24
3287	73.15
3286	73.05
3285	72.94
3284	72.84
3283	72.73
3282	72.61
3281	72.49
3280	72.36
3279	72.22
3278	72.08
3277	71.93
3276	71.77
3275	71.61
3274	71.44
3273	71.27
3272	71.1
3271	70.93
3270	70.76
3269	70.58
3268	70.4
3267	70.24
3266	70.08
3265	69.94
3264	69.81
3263	69.7
3262	69.61
3261	69.53
3260	69.47
3259	69.43
3258	69.4
3257	69.4
3256	69.42
3255	69.46

3254	69.52
3253	69.61
3252	69.72
3251	69.84
3250	69.99
3249	70.14
3248	70.3
3247	70.47
3246	70.64
3245	70.81
3244	70.99
3243	71.18
3242	71.37
3241	71.54
3240	71.71
3239	71.87
3238	72.04
3237	72.2
3236	72.36
3235	72.52
3234	72.67
3233	72.8
3232	72.91
3231	73.01
3230	73.11
3229	73.2
3228	73.3
3227	73.4
3226	73.5
3225	73.59
3224	73.69
3223	73.8
3222	73.92
3221	74.05

3220	74.17
3219	74.29
3218	74.42
3217	74.55
3216	74.68
3215	74.83
3214	74.98
3213	75.13
3212	75.28
3211	75.42
3210	75.57
3209	75.71
3208	75.86
3207	76.02
3206	76.18
3205	76.33
3204	76.48
3203	76.63
3202	76.78
3201	76.92
3200	77.05
3199	77.18
3198	77.31
3197	77.44
3196	77.59
3195	77.74
3194	77.89
3193	78.03
3192	78.18
3191	78.32
3190	78.46
3189	78.59
3188	78.73
3187	78.87

3186	79.01
3185	79.15
3184	79.29
3183	79.43
3182	79.57
3181	79.72
3180	79.87
3179	80.01
3178	80.14
3177	80.26
3176	80.39
3175	80.52
3174	80.65
3173	80.78
3172	80.92
3171	81.06
3170	81.19
3169	81.31
3168	81.43
3167	81.54
3166	81.65
3165	81.76
3164	81.88
3163	81.99
3162	82.1
3161	82.21
3160	82.31
3159	82.42
3158	82.52
3157	82.62
3156	82.73
3155	82.83
3154	82.93
3153	83.02

3152	83.1
3151	83.16
3150	83.22
3149	83.28
3148	83.34
3147	83.41
3146	83.47
3145	83.53
3144	83.58
3143	83.64
3142	83.69
3141	83.74
3140	83.77
3139	83.79
3138	83.8
3137	83.81
3136	83.81
3135	83.8
3134	83.78
3133	83.76
3132	83.73
3131	83.7
3130	83.66
3129	83.6
3128	83.54
3127	83.47
3126	83.4
3125	83.34
3124	83.28
3123	83.23
3122	83.18
3121	83.12
3120	83.06
3119	82.99

3118	82.92
3117	82.85
3116	82.77
3115	82.69
3114	82.61
3113	82.55
3112	82.52
3111	82.52
3110	82.53
3109	82.56
3108	82.58
3107	82.6
3106	82.62
3105	82.66
3104	82.71
3103	82.79
3102	82.88
3101	82.97
3100	83.06
3099	83.15
3098	83.24
3097	83.35
3096	83.48
3095	83.62
3094	83.76
3093	83.9
3092	84.05
3091	84.2
3090	84.35
3089	84.5
3088	84.66
3087	84.83
3086	85.01
3085	85.18

3084	85.36
3083	85.55
3082	85.74
3081	85.93
3080	86.12
3079	86.3
3078	86.48
3077	86.65
3076	86.81
3075	86.98
3074	87.15
3073	87.34
3072	87.53
3071	87.72
3070	87.9
3069	88.07
3068	88.23
3067	88.38
3066	88.53
3065	88.67
3064	88.81
3063	88.96
3062	89.1
3061	89.25
3060	89.39
3059	89.53
3058	89.66
3057	89.79
3056	89.92
3055	90.04
3054	90.15
3053	90.26
3052	90.37
3051	90.46

3050	90.56
3049	90.65
3048	90.75
3047	90.85
3046	90.95
3045	91.03
3044	91.11
3043	91.2
3042	91.29
3041	91.38
3040	91.46
3039	91.54
3038	91.61
3037	91.68
3036	91.75
3035	91.82
3034	91.89
3033	91.94
3032	91.99
3031	92.04
3030	92.08
3029	92.13
3028	92.19
3027	92.25
3026	92.3
3025	92.36
3024	92.41
3023	92.46
3022	92.52
3021	92.56
3020	92.61
3019	92.65
3018	92.69
3017	92.73

3016	92.78
3015	92.83
3014	92.87
3013	92.93
3012	92.99
3011	93.06
3010	93.14
3009	93.21
3008	93.28
3007	93.34
3006	93.4
3005	93.45
3004	93.51
3003	93.57
3002	93.64
3001	93.72
3000	93.81
2999	93.92
2998	94.04
2997	94.15
2996	94.25
2995	94.33
2994	94.4
2993	94.45
2992	94.49
2991	94.53
2990	94.56
2989	94.59
2988	94.62
2987	94.67
2986	94.73
2985	94.81
2984	94.9
2983	94.98

2982	95.02
2981	95.02
2980	94.98
2979	94.91
2978	94.82
2977	94.74
2976	94.67
2975	94.62
2974	94.57
2973	94.5
2972	94.42
2971	94.31
2970	94.15
2969	93.95
2968	93.71
2967	93.46
2966	93.21
2965	92.97
2964	92.77
2963	92.62
2962	92.53
2961	92.49
2960	92.51
2959	92.57
2958	92.66
2957	92.78
2956	92.9
2955	93.01
2954	93.11
2953	93.19
2952	93.26
2951	93.32
2950	93.37
2949	93.4

2948	93.4
2947	93.37
2946	93.29
2945	93.18
2944	93.03
2943	92.86
2942	92.67
2941	92.46
2940	92.24
2939	92.03
2938	91.83
2937	91.64
2936	91.43
2935	91.21
2934	90.95
2933	90.67
2932	90.38
2931	90.09
2930	89.79
2929	89.49
2928	89.18
2927	88.87
2926	88.55
2925	88.24
2924	87.93
2923	87.64
2922	87.37
2921	87.13
2920	86.95
2919	86.88
2918	86.94
2917	87.17
2916	87.54
2915	88.01

2914	88.53
2913	89.02
2912	89.44
2911	89.79
2910	90.08
2909	90.31
2908	90.51
2907	90.68
2906	90.83
2905	90.96
2904	91.06
2903	91.14
2902	91.19
2901	91.19
2900	91.16
2899	91.09
2898	91
2897	90.89
2896	90.78
2895	90.68
2894	90.62
2893	90.58
2892	90.58
2891	90.62
2890	90.69
2889	90.78
2888	90.86
2887	90.93
2886	90.97
2885	90.98
2884	90.98
2883	90.96
2882	90.93
2881	90.9

2880	90.88
2879	90.86
2878	90.86
2877	90.88
2876	90.91
2875	90.96
2874	91.01
2873	91.06
2872	91.12
2871	91.18
2870	91.26
2869	91.35
2868	91.45
2867	91.54
2866	91.62
2865	91.69
2864	91.74
2863	91.78
2862	91.79
2861	91.78
2860	91.73
2859	91.65
2858	91.53
2857	91.39
2856	91.2
2855	90.96
2854	90.68
2853	90.41
2852	90.2
2851	90.13
2850	90.25
2849	90.6
2848	91.12
2847	91.73

2846	92.35
2845	92.91
2844	93.4
2843	93.81
2842	94.15
2841	94.44
2840	94.68
2839	94.88
2838	95.04
2837	95.17
2836	95.3
2835	95.42
2834	95.54
2833	95.66
2832	95.78
2831	95.89
2830	95.98
2829	96.06
2828	96.12
2827	96.16
2826	96.19
2825	96.21
2824	96.24
2823	96.28
2822	96.32
2821	96.36
2820	96.4
2819	96.45
2818	96.5
2817	96.56
2816	96.62
2815	96.69
2814	96.75
2813	96.8

2812	96.84
2811	96.89
2810	96.93
2809	96.98
2808	97.02
2807	97.08
2806	97.13
2805	97.19
2804	97.24
2803	97.29
2802	97.34
2801	97.4
2800	97.46
2799	97.51
2798	97.56
2797	97.61
2796	97.66
2795	97.71
2794	97.76
2793	97.81
2792	97.87
2791	97.91
2790	97.96
2789	98.01
2788	98.06
2787	98.1
2786	98.13
2785	98.17
2784	98.19
2783	98.21
2782	98.22
2781	98.22
2780	98.22
2779	98.21

2778	98.22
2777	98.22
2776	98.23
2775	98.22
2774	98.22
2773	98.21
2772	98.21
2771	98.21
2770	98.21
2769	98.2
2768	98.19
2767	98.19
2766	98.2
2765	98.2
2764	98.21
2763	98.22
2762	98.23
2761	98.25
2760	98.26
2759	98.29
2758	98.3
2757	98.32
2756	98.33
2755	98.35
2754	98.37
2753	98.4
2752	98.43
2751	98.45
2750	98.46
2749	98.48
2748	98.49
2747	98.51
2746	98.52
2745	98.53

2744	98.53
2743	98.54
2742	98.54
2741	98.55
2740	98.56
2739	98.57
2738	98.57
2737	98.56
2736	98.55
2735	98.55
2734	98.55
2733	98.56
2732	98.56
2731	98.57
2730	98.58
2729	98.6
2728	98.61
2727	98.62
2726	98.62
2725	98.62
2724	98.62
2723	98.62
2722	98.62
2721	98.62
2720	98.62
2719	98.63
2718	98.64
2717	98.65
2716	98.67
2715	98.68
2714	98.69
2713	98.7
2712	98.71
2711	98.71

2710	98.72
2709	98.74
2708	98.75
2707	98.75
2706	98.76
2705	98.76
2704	98.77
2703	98.78
2702	98.8
2701	98.81
2700	98.83
2699	98.84
2698	98.86
2697	98.88
2696	98.89
2695	98.89
2694	98.89
2693	98.88
2692	98.88
2691	98.89
2690	98.89
2689	98.89
2688	98.9
2687	98.91
2686	98.92
2685	98.93
2684	98.94
2683	98.95
2682	98.95
2681	98.96
2680	98.97
2679	98.99
2678	98.99
2677	99

2676	98.99
2675	98.99
2674	98.99
2673	98.99
2672	99
2671	99
2670	99
2669	99
2668	98.99
2667	98.99
2666	98.99
2665	98.99
2664	98.98
2663	98.97
2662	98.96
2661	98.96
2660	98.95
2659	98.95
2658	98.96
2657	98.97
2656	98.97
2655	98.97
2654	98.97
2653	98.98
2652	98.98
2651	98.98
2650	98.98
2649	98.97
2648	98.96
2647	98.95
2646	98.94
2645	98.94
2644	98.94
2643	98.95

2642	98.97
2641	98.98
2640	99
2639	99.01
2638	99.01
2637	99.01
2636	99.01
2635	99.02
2634	99.02
2633	99.02
2632	99.02
2631	99.01
2630	99.01
2629	99.01
2628	99.03
2627	99.04
2626	99.05
2625	99.06
2624	99.07
2623	99.07
2622	99.08
2621	99.08
2620	99.08
2619	99.09
2618	99.1
2617	99.1
2616	99.1
2615	99.09
2614	99.09
2613	99.09
2612	99.09
2611	99.09
2610	99.1
2609	99.11

2608	99.11
2607	99.12
2606	99.12
2605	99.12
2604	99.13
2603	99.14
2602	99.14
2601	99.15
2600	99.15
2599	99.16
2598	99.17
2597	99.18
2596	99.18
2595	99.18
2594	99.17
2593	99.16
2592	99.16
2591	99.17
2590	99.18
2589	99.18
2588	99.18
2587	99.18
2586	99.18
2585	99.17
2584	99.16
2583	99.16
2582	99.17
2581	99.17
2580	99.18
2579	99.2
2578	99.22
2577	99.23
2576	99.24
2575	99.24

2574	99.24
2573	99.24
2572	99.25
2571	99.26
2570	99.26
2569	99.26
2568	99.25
2567	99.25
2566	99.24
2565	99.24
2564	99.25
2563	99.26
2562	99.28
2561	99.3
2560	99.31
2559	99.32
2558	99.34
2557	99.34
2556	99.35
2555	99.36
2554	99.37
2553	99.38
2552	99.38
2551	99.39
2550	99.4
2549	99.41
2548	99.43
2547	99.42
2546	99.41
2545	99.39
2544	99.37
2543	99.36
2542	99.36
2541	99.35

2540	99.33
2539	99.32
2538	99.33
2537	99.35
2536	99.38
2535	99.4
2534	99.41
2533	99.41
2532	99.39
2531	99.37
2530	99.36
2529	99.36
2528	99.37
2527	99.37
2526	99.37
2525	99.36
2524	99.35
2523	99.34
2522	99.33
2521	99.32
2520	99.31
2519	99.32
2518	99.34
2517	99.35
2516	99.35
2515	99.34
2514	99.34
2513	99.35
2512	99.35
2511	99.36
2510	99.37
2509	99.38
2508	99.38
2507	99.38

2506	99.38
2505	99.39
2504	99.4
2503	99.41
2502	99.41
2501	99.4
2500	99.39
2499	99.39
2498	99.39
2497	99.4
2496	99.41
2495	99.41
2494	99.42
2493	99.43
2492	99.43
2491	99.43
2490	99.43
2489	99.42
2488	99.42
2487	99.42
2486	99.42
2485	99.43
2484	99.43
2483	99.42
2482	99.42
2481	99.43
2480	99.45
2479	99.46
2478	99.45
2477	99.44
2476	99.43
2475	99.42
2474	99.4
2473	99.38

2472	99.37
2471	99.38
2470	99.41
2469	99.44
2468	99.44
2467	99.44
2466	99.43
2465	99.42
2464	99.43
2463	99.43
2462	99.44
2461	99.45
2460	99.45
2459	99.43
2458	99.42
2457	99.42
2456	99.42
2455	99.41
2454	99.4
2453	99.38
2452	99.38
2451	99.39
2450	99.41
2449	99.42
2448	99.4
2447	99.39
2446	99.38
2445	99.38
2444	99.4
2443	99.4
2442	99.39
2441	99.38
2440	99.37
2439	99.38

2438	99.4
2437	99.42
2436	99.43
2435	99.42
2434	99.41
2433	99.4
2432	99.41
2431	99.42
2430	99.43
2429	99.43
2428	99.42
2427	99.41
2426	99.4
2425	99.4
2424	99.39
2423	99.4
2422	99.41
2421	99.42
2420	99.42
2419	99.42
2418	99.4
2417	99.39
2416	99.38
2415	99.38
2414	99.4
2413	99.41
2412	99.41
2411	99.41
2410	99.41
2409	99.41
2408	99.42
2407	99.43
2406	99.44
2405	99.43

2404	99.42
2403	99.42
2402	99.41
2401	99.39
2400	99.38
2399	99.38
2398	99.39
2397	99.4
2396	99.42
2395	99.44
2394	99.45
2393	99.44
2392	99.42
2391	99.4
2390	99.39
2389	99.39
2388	99.38
2387	99.38
2386	99.38
2385	99.4
2384	99.42
2383	99.44
2382	99.44
2381	99.42
2380	99.41
2379	99.41
2378	99.41
2377	99.42
2376	99.42
2375	99.42
2374	99.42
2373	99.44
2372	99.48
2371	99.53

2370	99.56
2369	99.55
2368	99.51
2367	99.46
2366	99.42
2365	99.37
2364	99.33
2363	99.31
2362	99.31
2361	99.3
2360	99.31
2359	99.32
2358	99.34
2357	99.34
2356	99.34
2355	99.32
2354	99.31
2353	99.3
2352	99.28
2351	99.28
2350	99.26
2349	99.25
2348	99.24
2347	99.23
2346	99.2
2345	99.18
2344	99.16
2343	99.15
2342	99.14
2341	99.14
2340	99.12
2339	99.09
2338	99.08
2337	99.08

2336	99.1
2335	99.12
2334	99.13
2333	99.12
2332	99.1
2331	99.08
2330	99.06
2329	99.02
2328	98.96
2327	98.87
2326	98.76
2325	98.68
2324	98.65
2323	98.69
2322	98.75
2321	98.81
2320	98.85
2319	98.87
2318	98.88
2317	98.9
2316	98.92
2315	98.93
2314	98.94
2313	98.94
2312	98.93
2311	98.93
2310	98.94
2309	98.95
2308	98.94
2307	98.92
2306	98.9
2305	98.89
2304	98.88
2303	98.88

2302	98.88
2301	98.89
2300	98.89
2299	98.89
2298	98.89
2297	98.9
2296	98.9
2295	98.89
2294	98.88
2293	98.86
2292	98.83
2291	98.81
2290	98.79
2289	98.77
2288	98.76
2287	98.76
2286	98.78
2285	98.81
2284	98.84
2283	98.86
2282	98.87
2281	98.86
2280	98.85
2279	98.84
2278	98.84
2277	98.85
2276	98.87
2275	98.88
2274	98.87
2273	98.87
2272	98.88
2271	98.91
2270	98.94
2269	98.94

2268	98.92
2267	98.91
2266	98.91
2265	98.93
2264	98.93
2263	98.92
2262	98.89
2261	98.87
2260	98.86
2259	98.88
2258	98.89
2257	98.9
2256	98.92
2255	98.93
2254	98.95
2253	98.96
2252	98.96
2251	98.97
2250	98.98
2249	98.99
2248	99
2247	99
2246	98.99
2245	98.97
2244	98.94
2243	98.91
2242	98.88
2241	98.84
2240	98.8
2239	98.78
2238	98.81
2237	98.88
2236	98.96
2235	99.03

2234	99.08
2233	99.1
2232	99.1
2231	99.1
2230	99.1
2229	99.12
2228	99.14
2227	99.13
2226	99.09
2225	99.05
2224	99.02
2223	99
2222	98.98
2221	98.96
2220	98.95
2219	98.96
2218	98.99
2217	99.03
2216	99.05
2215	99.05
2214	99.04
2213	99
2212	98.96
2211	98.94
2210	98.92
2209	98.89
2208	98.84
2207	98.81
2206	98.8
2205	98.79
2204	98.79
2203	98.79
2202	98.8
2201	98.82

2200	98.85
2199	98.85
2198	98.81
2197	98.75
2196	98.73
2195	98.76
2194	98.8
2193	98.81
2192	98.79
2191	98.76
2190	98.75
2189	98.73
2188	98.68
2187	98.62
2186	98.58
2185	98.59
2184	98.65
2183	98.75
2182	98.86
2181	98.94
2180	98.97
2179	98.97
2178	98.93
2177	98.86
2176	98.81
2175	98.78
2174	98.77
2173	98.76
2172	98.75
2171	98.77
2170	98.8
2169	98.83
2168	98.82
2167	98.78

2166	98.71
2165	98.65
2164	98.64
2163	98.69
2162	98.79
2161	98.98
2160	99.3
2159	99.69
2158	99.97
2157	100.01
2156	99.83
2155	99.62
2154	99.49
2153	99.46
2152	99.49
2151	99.53
2150	99.56
2149	99.56
2148	99.52
2147	99.46
2146	99.39
2145	99.34
2144	99.33
2143	99.35
2142	99.39
2141	99.42
2140	99.43
2139	99.41
2138	99.38
2137	99.36
2136	99.4
2135	99.45
2134	99.47
2133	99.41

2132	99.3
2131	99.19
2130	99.11
2129	99.07
2128	99.05
2127	99.05
2126	99.04
2125	99.02
2124	98.99
2123	98.99
2122	99.01
2121	99.03
2120	99.02
2119	98.99
2118	98.94
2117	98.88
2116	98.81
2115	98.76
2114	98.73
2113	98.72
2112	98.72
2111	98.73
2110	98.75
2109	98.74
2108	98.71
2107	98.68
2106	98.67
2105	98.68
2104	98.69
2103	98.7
2102	98.7
2101	98.69
2100	98.68
2099	98.67

2098	98.67
2097	98.71
2096	98.79
2095	98.87
2094	98.9
2093	98.87
2092	98.81
2091	98.76
2090	98.75
2089	98.76
2088	98.78
2087	98.8
2086	98.81
2085	98.83
2084	98.85
2083	98.86
2082	98.85
2081	98.83
2080	98.81
2079	98.8
2078	98.79
2077	98.78
2076	98.77
2075	98.75
2074	98.77
2073	98.82
2072	98.89
2071	98.95
2070	98.95
2069	98.88
2068	98.8
2067	98.76
2066	98.79
2065	98.85

2064	98.92
2063	98.98
2062	99.05
2061	99.11
2060	99.18
2059	99.21
2058	99.2
2057	99.19
2056	99.2
2055	99.25
2054	99.29
2053	99.27
2052	99.21
2051	99.17
2050	99.19
2049	99.24
2048	99.27
2047	99.27
2046	99.25
2045	99.26
2044	99.3
2043	99.38
2042	99.45
2041	99.49
2040	99.5
2039	99.53
2038	99.56
2037	99.57
2036	99.61
2035	99.71
2034	99.83
2033	99.89
2032	99.87
2031	99.81

2030	99.79
2029	99.82
2028	99.85
2027	99.88
2026	99.92
2025	99.98
2024	100.06
2023	100.11
2022	100.1
2021	100.06
2020	100.02
2019	99.99
2018	99.93
2017	99.88
2016	99.89
2015	99.97
2014	100.07
2013	100.12
2012	100.1
2011	100.08
2010	100.06
2009	100.08
2008	100.1
2007	100.12
2006	100.09
2005	100
2004	99.9
2003	99.85
2002	99.85
2001	99.85
2000	99.85
1999	99.84
1998	99.8
1997	99.72

1996	99.64
1995	99.6
1994	99.57
1993	99.51
1992	99.42
1991	99.36
1990	99.35
1989	99.34
1988	99.33
1987	99.33
1986	99.34
1985	99.35
1984	99.32
1983	99.25
1982	99.17
1981	99.1
1980	99.1
1979	99.2
1978	99.34
1977	99.49
1976	99.6
1975	99.68
1974	99.75
1973	99.81
1972	99.86
1971	99.91
1970	99.93
1969	99.92
1968	99.92
1967	99.91
1966	99.86
1965	99.77
1964	99.68
1963	99.66

1962	99.73
1961	99.84
1960	99.93
1959	99.97
1958	99.93
1957	99.84
1956	99.74
1955	99.64
1954	99.54
1953	99.44
1952	99.39
1951	99.42
1950	99.49
1949	99.55
1948	99.59
1947	99.62
1946	99.64
1945	99.63
1944	99.61
1943	99.62
1942	99.62
1941	99.58
1940	99.52
1939	99.47
1938	99.45
1937	99.44
1936	99.45
1935	99.48
1934	99.52
1933	99.56
1932	99.59
1931	99.61
1930	99.61
1929	99.6

1928	99.58
1927	99.58
1926	99.57
1925	99.53
1924	99.46
1923	99.4
1922	99.36
1921	99.34
1920	99.34
1919	99.38
1918	99.44
1917	99.49
1916	99.52
1915	99.56
1914	99.59
1913	99.6
1912	99.59
1911	99.54
1910	99.49
1909	99.46
1908	99.48
1907	99.53
1906	99.58
1905	99.58
1904	99.55
1903	99.51
1902	99.49
1901	99.51
1900	99.53
1899	99.55
1898	99.54
1897	99.51
1896	99.48
1895	99.45

1894	99.44
1893	99.43
1892	99.42
1891	99.43
1890	99.45
1889	99.48
1888	99.48
1887	99.47
1886	99.45
1885	99.44
1884	99.45
1883	99.47
1882	99.47
1881	99.44
1880	99.38
1879	99.33
1878	99.3
1877	99.27
1876	99.25
1875	99.25
1874	99.26
1873	99.28
1872	99.29
1871	99.3
1870	99.3
1869	99.28
1868	99.24
1867	99.21
1866	99.21
1865	99.24
1864	99.27
1863	99.31
1862	99.31
1861	99.29

1860	99.27
1859	99.26
1858	99.29
1857	99.32
1856	99.34
1855	99.35
1854	99.33
1853	99.32
1852	99.3
1851	99.29
1850	99.28
1849	99.25
1848	99.23
1847	99.21
1846	99.18
1845	99.16
1844	99.16
1843	99.21
1842	99.26
1841	99.27
1840	99.26
1839	99.25
1838	99.24
1837	99.23
1836	99.2
1835	99.18
1834	99.19
1833	99.22
1832	99.27
1831	99.31
1830	99.32
1829	99.31
1828	99.31
1827	99.32

1826	99.32
1825	99.28
1824	99.21
1823	99.16
1822	99.12
1821	99.1
1820	99.11
1819	99.14
1818	99.17
1817	99.18
1816	99.18
1815	99.17
1814	99.15
1813	99.13
1812	99.12
1811	99.12
1810	99.1
1809	99.07
1808	99.04
1807	99.03
1806	99.04
1805	99.06
1804	99.07
1803	99.08
1802	99.05
1801	99
1800	98.95
1799	98.91
1798	98.89
1797	98.88
1796	98.88
1795	98.89
1794	98.89
1793	98.87

1792	98.85
1791	98.85
1790	98.85
1789	98.85
1788	98.84
1787	98.83
1786	98.81
1785	98.79
1784	98.78
1783	98.79
1782	98.8
1781	98.79
1780	98.76
1779	98.74
1778	98.72
1777	98.72
1776	98.73
1775	98.75
1774	98.76
1773	98.73
1772	98.67
1771	98.63
1770	98.59
1769	98.55
1768	98.53
1767	98.53
1766	98.54
1765	98.53
1764	98.51
1763	98.49
1762	98.47
1761	98.45
1760	98.43
1759	98.42

1758	98.4
1757	98.37
1756	98.35
1755	98.35
1754	98.36
1753	98.34
1752	98.29
1751	98.23
1750	98.19
1749	98.14
1748	98.1
1747	98.09
1746	98.1
1745	98.12
1744	98.14
1743	98.18
1742	98.21
1741	98.2
1740	98.18
1739	98.18
1738	98.2
1737	98.21
1736	98.21
1735	98.22
1734	98.26
1733	98.33
1732	98.36
1731	98.39
1730	98.46
1729	98.54
1728	98.64
1727	98.78
1726	98.99
1725	99.26

1724	99.6
1723	99.99
1722	100.36
1721	100.64
1720	100.74
1719	100.53
1718	99.89
1717	99.06
1716	98.38
1715	97.91
1714	97.59
1713	97.37
1712	97.18
1711	97.02
1710	96.86
1709	96.72
1708	96.59
1707	96.46
1706	96.32
1705	96.19
1704	96.09
1703	95.98
1702	95.83
1701	95.62
1700	95.42
1699	95.34
1698	95.33
1697	95.27
1696	95.12
1695	94.88
1694	94.64
1693	94.43
1692	94.26
1691	94.08

1690	93.87
1689	93.66
1688	93.52
1687	93.46
1686	93.38
1685	93
1684	92.25
1683	91.51
1682	90.91
1681	90.3
1680	89.63
1679	88.96
1678	88.3
1677	87.56
1676	86.62
1675	85.64
1674	84.88
1673	84.29
1672	83.69
1671	82.98
1670	82.14
1669	81.3
1668	80.58
1667	79.98
1666	79.44
1665	78.81
1664	78
1663	77.06
1662	76.17
1661	75.45
1660	74.86
1659	74.39
1658	74.06
1657	73.85

1656	73.75
1655	73.76
1654	73.95
1653	74.24
1652	74.4
1651	74.42
1650	74.42
1649	74.49
1648	74.57
1647	74.68
1646	74.83
1645	74.9
1644	74.86
1643	74.79
1642	74.72
1641	74.63
1640	74.5
1639	74.37
1638	74.23
1637	73.96
1636	73.5
1635	73.04
1634	72.68
1633	72.34
1632	71.97
1631	71.56
1630	71.12
1629	70.6
1628	70
1627	69.41
1626	68.87
1625	68.3
1624	67.63
1623	66.97

1622	66.54
1621	66.34
1620	66.27
1619	66.4
1618	66.88
1617	67.84
1616	69.03
1615	70.15
1614	71.19
1613	72.29
1612	73.5
1611	74.78
1610	76.04
1609	77.24
1608	78.33
1607	79.27
1606	80.06
1605	80.74
1604	81.34
1603	81.86
1602	82.3
1601	82.67
1600	82.97
1599	83.19
1598	83.31
1597	83.37
1596	83.38
1595	83.32
1594	83.19
1593	83.01
1592	82.78
1591	82.48
1590	82.11
1589	81.7

1588	81.24
1587	80.73
1586	80.15
1585	79.52
1584	78.88
1583	78.22
1582	77.54
1581	76.88
1580	76.27
1579	75.7
1578	75.1
1577	74.35
1576	73.5
1575	72.8
1574	72.3
1573	71.91
1572	71.58
1571	71.22
1570	70.86
1569	70.53
1568	70.26
1567	70
1566	69.69
1565	69.32
1564	68.94
1563	68.63
1562	68.4
1561	68.18
1560	67.68
1559	66.73
1558	66.01
1557	65.71
1556	65.49
1555	65.41

1554	65.43
1553	65.56
1552	65.75
1551	66.04
1550	66.47
1549	66.93
1548	67.31
1547	67.67
1546	68.07
1545	68.54
1544	69
1543	69.41
1542	69.92
1541	70.72
1540	71.85
1539	72.82
1538	73.38
1537	73.71
1536	74.06
1535	74.62
1534	75.49
1533	76.47
1532	77.23
1531	77.74
1530	78.19
1529	78.65
1528	79.11
1527	79.55
1526	79.97
1525	80.35
1524	80.67
1523	81.01
1522	81.45
1521	81.91

1520	82.23
1519	82.4
1518	82.58
1517	82.8
1516	83.02
1515	83.2
1514	83.36
1513	83.54
1512	83.72
1511	83.92
1510	84.14
1509	84.35
1508	84.57
1507	84.91
1506	85.23
1505	85.31
1504	85.25
1503	85.21
1502	85.24
1501	85.34
1500	85.46
1499	85.62
1498	85.8
1497	85.97
1496	86.1
1495	86.17
1494	86.21
1493	86.24
1492	86.27
1491	86.31
1490	86.36
1489	86.45
1488	86.53
1487	86.56

1486	86.59
1485	86.6
1484	86.57
1483	86.52
1482	86.46
1481	86.39
1480	86.32
1479	86.27
1478	86.22
1477	86.15
1476	86.05
1475	85.93
1474	85.82
1473	85.74
1472	85.74
1471	85.75
1470	85.72
1469	85.67
1468	85.62
1467	85.56
1466	85.52
1465	85.49
1464	85.49
1463	85.5
1462	85.52
1461	85.53
1460	85.55
1459	85.52
1458	85.39
1457	85.25
1456	85.19
1455	85.16
1454	85.08
1453	84.99

1452	84.88
1451	84.73
1450	84.55
1449	84.32
1448	84.07
1447	83.84
1446	83.66
1445	83.51
1444	83.36
1443	83.22
1442	83.07
1441	82.91
1440	82.74
1439	82.56
1438	82.32
1437	81.99
1436	81.68
1435	81.45
1434	81.3
1433	81.17
1432	81.09
1431	81.07
1430	81.12
1429	81.2
1428	81.27
1427	81.35
1426	81.43
1425	81.49
1424	81.51
1423	81.52
1422	81.53
1421	81.51
1420	81.44
1419	81.34

1418	81.23
1417	81.14
1416	81.1
1415	81.16
1414	81.32
1413	81.56
1412	81.87
1411	82.26
1410	82.68
1409	83.07
1408	83.42
1407	83.73
1406	83.99
1405	84.18
1404	84.3
1403	84.37
1402	84.41
1401	84.42
1400	84.42
1399	84.41
1398	84.42
1397	84.43
1396	84.44
1395	84.43
1394	84.37
1393	84.24
1392	84.01
1391	83.7
1390	83.27
1389	82.67
1388	81.78
1387	80.65
1386	79.42
1385	78.16

1384	76.85
1383	75.53
1382	74.31
1381	73.3
1380	72.52
1379	72
1378	71.76
1377	71.78
1376	72.03
1375	72.5
1374	73.12
1373	73.73
1372	74.24
1371	74.7
1370	75.2
1369	75.75
1368	76.36
1367	77
1366	77.69
1365	78.45
1364	79.33
1363	80.33
1362	81.37
1361	82.32
1360	83.13
1359	83.84
1358	84.5
1357	85.1
1356	85.64
1355	86.12
1354	86.55
1353	86.94
1352	87.29
1351	87.64

1350	87.97
1349	88.29
1348	88.59
1347	88.85
1346	89.05
1345	89.19
1344	89.25
1343	89.25
1342	89.16
1341	88.98
1340	88.67
1339	88.27
1338	87.82
1337	87.3
1336	86.71
1335	86.06
1334	85.33
1333	84.53
1332	83.67
1331	82.82
1330	82.02
1329	81.33
1328	80.76
1327	80.33
1326	79.99
1325	79.71
1324	79.45
1323	79.19
1322	78.92
1321	78.62
1320	78.3
1319	77.99
1318	77.71
1317	77.45

1316	77.2
1315	76.97
1314	76.76
1313	76.57
1312	76.4
1311	76.29
1310	76.23
1309	76.23
1308	76.32
1307	76.51
1306	76.8
1305	77.15
1304	77.52
1303	77.92
1302	78.32
1301	78.72
1300	79.15
1299	79.63
1298	80.21
1297	80.86
1296	81.57
1295	82.29
1294	82.99
1293	83.62
1292	84.18
1291	84.68
1290	85.14
1289	85.6
1288	86.07
1287	86.56
1286	87.06
1285	87.54
1284	87.99
1283	88.39

1282	88.75
1281	89.05
1280	89.3
1279	89.51
1278	89.69
1277	89.83
1276	89.93
1275	89.97
1274	89.93
1273	89.82
1272	89.62
1271	89.35
1270	89.01
1269	88.58
1268	88.05
1267	87.42
1266	86.75
1265	86.1
1264	85.53
1263	85.13
1262	84.93
1261	84.9
1260	85.03
1259	85.25
1258	85.52
1257	85.83
1256	86.17
1255	86.53
1254	86.91
1253	87.3
1252	87.69
1251	88.06
1250	88.4
1249	88.68

1248	88.92
1247	89.11
1246	89.26
1245	89.37
1244	89.45
1243	89.51
1242	89.53
1241	89.53
1240	89.5
1239	89.44
1238	89.37
1237	89.31
1236	89.27
1235	89.25
1234	89.28
1233	89.32
1232	89.39
1231	89.47
1230	89.58
1229	89.72
1228	89.87
1227	90.02
1226	90.16
1225	90.29
1224	90.41
1223	90.52
1222	90.63
1221	90.75
1220	90.89
1219	91.02
1218	91.13
1217	91.18
1216	91.16
1215	91.06

1214	90.87
1213	90.59
1212	90.22
1211	89.78
1210	89.32
1209	88.85
1208	88.42
1207	88.05
1206	87.8
1205	87.67
1204	87.69
1203	87.85
1202	88.13
1201	88.49
1200	88.91
1199	89.36
1198	89.85
1197	90.37
1196	90.95
1195	91.58
1194	92.24
1193	92.89
1192	93.51
1191	94.06
1190	94.52
1189	94.87
1188	95.1
1187	95.19
1186	95.16
1185	95.02
1184	94.77
1183	94.46
1182	94.1
1181	93.74

1180	93.38
1179	93.05
1178	92.78
1177	92.57
1176	92.41
1175	92.27
1174	92.14
1173	92.02
1172	91.89
1171	91.73
1170	91.51
1169	91.18
1168	90.7
1167	90.08
1166	89.29
1165	88.34
1164	87.22
1163	85.95
1162	84.56
1161	83.1
1160	81.66
1159	80.31
1158	79.1
1157	78.07
1156	77.24
1155	76.64
1154	76.33
1153	76.29
1152	76.53
1151	77.04
1150	77.78
1149	78.68
1148	79.67
1147	80.67

1146	81.63
1145	82.5
1144	83.24
1143	83.84
1142	84.29
1141	84.59
1140	84.76
1139	84.82
1138	84.84
1137	84.83
1136	84.79
1135	84.71
1134	84.56
1133	84.28
1132	83.85
1131	83.24
1130	82.49
1129	81.62
1128	80.67
1127	79.73
1126	78.85
1125	78.07
1124	77.36
1123	76.7
1122	76.06
1121	75.41
1120	74.76
1119	74.14
1118	73.59
1117	73.13
1116	72.78
1115	72.58
1114	72.55
1113	72.7

1112	73.04
1111	73.55
1110	74.17
1109	74.83
1108	75.46
1107	76.06
1106	76.64
1105	77.22
1104	77.81
1103	78.43
1102	79.05
1101	79.64
1100	80.18
1099	80.66
1098	81.04
1097	81.26
1096	81.28
1095	81.08
1094	80.7
1093	80.18
1092	79.61
1091	79.05
1090	78.5
1089	77.94
1088	77.32
1087	76.6
1086	75.76
1085	74.78
1084	73.6
1083	72.25
1082	70.76
1081	69.17
1080	67.53
1079	65.95

1078	64.5
1077	63.22
1076	62.14
1075	61.27
1074	60.63
1073	60.19
1072	59.93
1071	59.83
1070	59.85
1069	59.98
1068	60.18
1067	60.48
1066	60.85
1065	61.23
1064	61.6
1063	61.97
1062	62.37
1061	62.79
1060	63.27
1059	63.88
1058	64.64
1057	65.54
1056	66.54
1055	67.57
1054	68.57
1053	69.5
1052	70.35
1051	71.16
1050	71.92
1049	72.57
1048	73.04
1047	73.3
1046	73.34
1045	73.15

1044	72.75
1043	72.15
1042	71.39
1041	70.48
1040	69.47
1039	68.4
1038	67.33
1037	66.26
1036	65.2
1035	64.2
1034	63.28
1033	62.42
1032	61.6
1031	60.81
1030	60.03
1029	59.28
1028	58.59
1027	58.04
1026	57.64
1025	57.37
1024	57.26
1023	57.3
1022	57.47
1021	57.68
1020	57.84
1019	57.9
1018	57.84
1017	57.65
1016	57.37
1015	57.08
1014	56.84
1013	56.65
1012	56.56
1011	56.61

1010	56.85
1009	57.28
1008	57.92
1007	58.78
1006	59.78
1005	60.85
1004	61.95
1003	63.09
1002	64.27
1001	65.45
1000	66.62
999	67.76
998	68.86
997	69.92
996	70.96
995	71.99
994	73.01
993	73.96
992	74.79
991	75.51
990	76.15
989	76.77
988	77.41
987	78.08
986	78.79
985	79.45
984	80.03
983	80.48
982	80.82
981	81.05
980	81.2
979	81.26
978	81.2
977	81.01

976	80.74
975	80.45
974	80.22
973	80.11
972	80.17
971	80.42
970	80.84
969	81.39
968	82.02
967	82.67
966	83.29
965	83.8
964	84.16
963	84.33
962	84.25
961	83.82
960	83
959	81.81
958	80.37
957	78.88
956	77.5
955	76.38
954	75.62
953	75.24
952	75.26
951	75.66
950	76.36
949	77.22
948	78.15
947	79.06
946	79.93
945	80.74
944	81.48
943	82.16

942	82.79
941	83.38
940	83.95
939	84.48
938	84.98
937	85.44
936	85.86
935	86.25
934	86.59
933	86.88
932	87.1
931	87.23
930	87.27
929	87.24
928	87.16
927	87.04
926	86.9
925	86.7
924	86.43
923	86.1
922	85.75
921	85.42
920	85.15
919	85
918	84.98
917	85.06
916	85.22
915	85.4
914	85.59
913	85.74
912	85.81
911	85.78
910	85.65
909	85.43

908	85.19
907	84.95
906	84.74
905	84.52
904	84.25
903	83.94
902	83.63
901	83.35
900	83.14
899	82.99
898	82.88
897	82.77
896	82.65
895	82.55
894	82.5
893	82.54
892	82.66
891	82.86
890	83.15
889	83.49
888	83.86
887	84.2
886	84.49
885	84.73
884	84.95
883	85.18
882	85.4
881	85.58
880	85.69
879	85.72
878	85.71
877	85.68
876	85.66
875	85.65

874	85.64
873	85.65
872	85.67
871	85.68
870	85.68
869	85.67
868	85.66
867	85.66
866	85.65
865	85.59
864	85.46
863	85.31
862	85.18
861	85.08
860	85.01
859	84.97
858	84.93
857	84.86
856	84.74
855	84.6
854	84.48
853	84.38
852	84.3
851	84.25
850	84.22
849	84.2
848	84.15
847	84.07
846	83.99
845	83.92
844	83.86
843	83.8
842	83.74
841	83.67

840	83.6
839	83.56
838	83.56
837	83.61
836	83.66
835	83.7
834	83.68
833	83.57
832	83.38
831	83.14
830	82.89
829	82.65
828	82.42
827	82.23
826	82.1
825	82
824	81.89
823	81.76
822	81.61
821	81.48
820	81.36
819	81.27
818	81.21
817	81.15
816	81.07
815	81
814	80.94
813	80.89
812	80.84
811	80.79
810	80.73
809	80.68
808	80.62
807	80.54

806	80.45
805	80.33
804	80.19
803	80.06
802	79.93
801	79.81
800	79.71
799	79.62
798	79.54
797	79.47
796	79.35
795	79.18
794	78.97
793	78.75
792	78.54
791	78.34
790	78.16
789	77.99
788	77.81
787	77.65
786	77.5
785	77.36
784	77.21
783	77.05
782	76.87
781	76.67
780	76.47
779	76.29
778	76.11
777	75.91
776	75.69
775	75.46
774	75.23
773	74.98

772	74.71
771	74.44
770	74.18
769	73.93
768	73.69
767	73.45
766	73.22
765	72.98
764	72.72
763	72.47
762	72.26
761	72.07
760	71.9
759	71.73
758	71.57
757	71.4
756	71.23
755	71.06
754	70.9
753	70.75
752	70.59
751	70.46
750	70.36
749	70.27
748	70.17
747	70.08
746	69.99
745	69.91
744	69.82
743	69.75
742	69.7
741	69.68
740	69.65
739	69.63

738	69.62
737	69.63
736	69.64
735	69.66
734	69.67
733	69.64
732	69.57
731	69.47
730	69.35
729	69.22
728	69.07
727	68.93
726	68.81
725	68.69
724	68.56
723	68.4
722	68.22
721	68.05
720	67.91
719	67.81
718	67.76
717	67.7
716	67.61
715	67.49
714	67.35
713	67.19
712	67.02
711	66.86
710	66.73
709	66.61
708	66.47
707	66.29
706	66.08
705	65.85

704	65.64
703	65.51
702	65.47
701	65.49
700	65.53
699	65.6
698	65.67
697	65.68
696	65.63
695	65.53
694	65.4
693	65.24
692	65.07
691	64.92
690	64.83
689	64.77
688	64.73
687	64.73
686	64.77
685	64.83
684	64.89
683	64.96
682	65.07
681	65.2
680	65.35
679	65.48
678	65.61
677	65.74
676	65.86
675	65.99
674	66.12
673	66.25
672	66.35
671	66.47

670	66.64
669	66.82
668	66.87
667	66.75
666	66.59
665	66.46
664	66.38
663	66.31
662	66.25
661	66.16
660	66.06
659	65.98
658	65.94
657	65.91
656	65.85
655	65.77
654	65.67
653	65.56
652	65.43
651	65.32
650	65.28
649	65.26
648	65.26
647	65.23
646	65.16
645	65.02
644	64.82
643	64.6
642	64.35
641	63.98
640	63.48
639	62.92
638	62.42
637	62.02

636	61.68
635	61.39
634	61.15
633	60.95
632	60.82
631	60.77
630	60.77
629	60.72
628	60.61
627	60.48
626	60.39
625	60.33
624	60.26
623	60.16
622	60.03
621	59.85
620	59.64
619	59.45
618	59.32
617	59.2
616	59.06
615	58.96
614	58.91
613	58.89
612	58.87
611	58.86
610	58.87
609	58.87
608	58.83
607	58.78
606	58.71
605	58.58
604	58.37
603	58.15
