# Supplementary figures and images for: Biotechnology-driven extraction and characterisation of Chitosan from the West African river prawn (Macrobrachium vollenhovenii) and American Cockroach (Periplaneta americana) using a modified approach
Source: PLoS One. 2026 May 28;21(5):e0349133. doi: 10.1371/journal.pone.0349133 (PMC13218523; doi:10.1371/journal.pone.0349133)

|           | Chitin Yield | Chitosan Yi | DD% of Chitosan |
|-----------|--------------|-------------|-----------------|
| Prawn     | 29.53        | 28.13       | 68.79           |
| Cockroach | 17.78        | 11.56       | 81.21           |

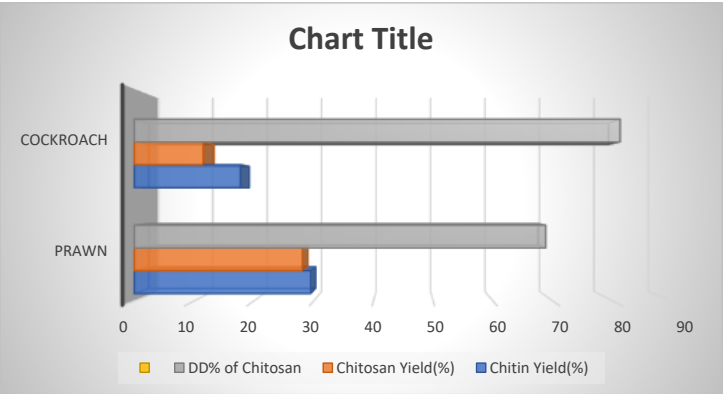

Supplement: S1 File — (ZIP) [file pone.0349133.s001.zip › Results ANalysis.pdf]
